# Not the Silver Bullet: Uncovering the Unexpected Limited Impacts of Silver-Containing Showerheads on the Drinking Water Microbiome

**DOI:** 10.1021/acsestwater.4c00492

**Published:** 2024-11-25

**Authors:** Sarah Pitell, Isaiah Spencer-Williams, Daniel Huffman, Paige Moncure, Jill Millstone, Janet Stout, Leanne Gilbertson, Sarah-Jane Haig

**Affiliations:** † Department of Civil and Environmental Engineering, University of Pittsburgh, Pittsburgh, Pennsylvania 15261, United States; ‡ Department of Chemistry, University of Pittsburgh, Pittsburgh, Pennsylvania 15213, United States; § Department of Mechanical Engineering and Materials Science, University of Pittsburgh, Pittsburgh, Pennsylvania 15261, United States; ∥ Department of Chemical and Petroleum Engineering, University of Pittsburgh, Pittsburgh, Pennsylvania 15261, United States; ⊥ Special Pathogens Laboratory, Pittsburgh, Pennsylvania 15219, United States; # Department of Environmental & Occupational Health, University of Pittsburgh, Pittsburgh, Pennsylvania 15261, United States; ¶ Department of Civil and Environmental Engineering, Duke University, Durham, North Carolina 27708, United States

**Keywords:** opportunistic pathogen, DWPI, Legionella, NTM, drinking water, 16S rRNA, silver, shower, antimicrobial, point-of-use device

## Abstract

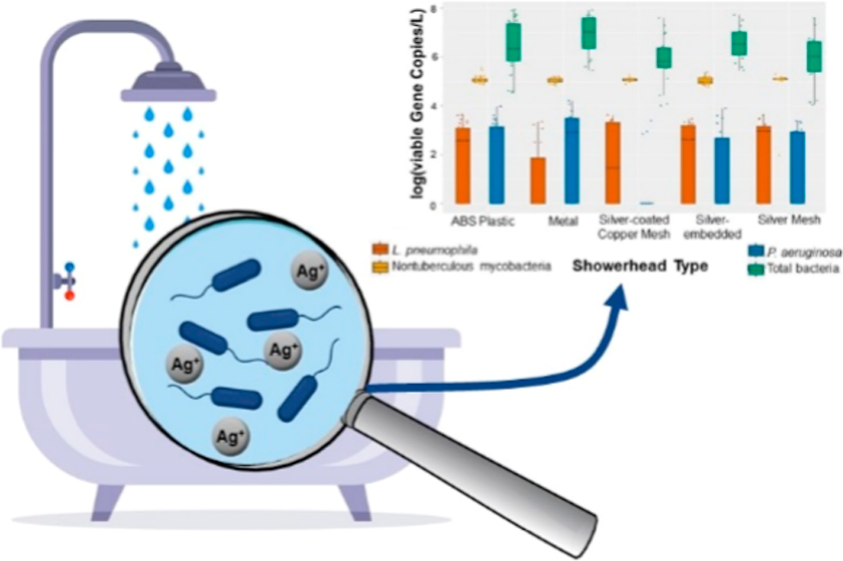

The incidence of waterborne disease outbreaks in the United States attributed to drinking water-associated pathogens that can cause infections in the immunocompromised DWPIs (e.g., *Legionella pneumophila*, nontuberculous mycobacteria (NTM), and *Pseudomonas aeruginosa*, among others) appears to be increasing. An emerging technology adopted to reduce DWPIs are point-of-use devices, such as showerheads that contain silver, a known antimicrobial material. In this study, we evaluate the effect of silver-containing showerheads on DWPI density and the broader microbiome in shower water under real-use conditions in a full-scale shower system, considering three different silver-modified showerhead designs: (i) silver mesh within the showerhead, (ii) silver-coated copper mesh in the head and hose, and (iii) silver-embedded polymer composite compared to conventional plastic and metal showerheads. We found no significant difference in targeted DWPI transcriptional activity in collected water across silver and nonsilver shower head designs. Yet, the presence of silver and how it was incorporated in the showerhead influenced the metal concentrations, microbial rare taxa, and microbiome functionality. Microbial dynamics were also influenced by the showerhead age (i.e., time after installation). The results of this study provide valuable information for consumers and building managers to consider when choosing a showerhead meant to reduce microorganisms in shower water.

## Abstract 2

Silver is a common additive in antimicrobial showerheads and was found to affect certain rare microbes and microbial community function within shower water but did not influence DWPI transcriptional activity.

## Introduction

In the United States, the number of clinical cases caused by drinking water-associated pathogens that cause infections in the immunocompromised (DWPIs)^1^ surpasses those caused by regulated fecal-borne pathogens in drinking water such as *Giardia spp* or *Cryptosporidium spp*.^2,3^ This trend is expected to persist with the rising incidence of DWPI infections,^4−6^ particularly among vulnerable populations such as the very young or old and those with immunosuppressive conditions.^7,8^ DWPIs such as *Legionella pneumophila*, *Pseudomonas aeruginosa*, and nontuberculous mycobacteria (NTM) cause respiratory illnesses that are often challenging and expensive to treat^9,10^ and, as such, cost the US economy $2.39 billion annually in direct health care costs.^2^ Today, the incidence of waterborne disease outbreaks in the United States attributed to DWPIs, which are not regulated by the US EPA (e.g., *L. pneumophila*, nontuberculous mycobacteria (NTM), and *P. aeruginosa*, among others) appears to be increasing. For example, the incidences of legionellosis and nontuberculous mycobacterial pulmonary disease increased 225% and 300% from 2000 to 2017, respectively. An emerging technology adopted to reduce DWPIs are antimicrobial point-of-use devices. Although DWPIs are commonly quantified in low numbers in the environment, they proliferate within drinking water distribution systems and building plumbing and are found in the biofilms that form in fixtures^11,12^ and pipes.^13−16^ Drinking water biofilms can influence the microbial composition of drinking water due to sloughing,^17^ and many factors such as plumbing material and age of the fixture itself^13−15,18^ can impact their composition. Because clinical isolates have been linked to DWPIs taken from patients’ homes,^19,20^ developing strategies to combat DWPI exposure is essential for reducing disease burden. When considering the potential routes of exposure to DWPIs, showering accounts for 20% of daily drinking water use for the average American^21^ and has many attributes that make it a likely source of DWPI infection: (i) showering occurs frequently for a majority of the population,^22^ (ii) showerheads generate abundant respirable bioaerosols that may contain DWPIs^23−25^ within the respirable range of the average adult’s nose and mouth, and (iii) DWPIs have been found to be enriched in showerheads and hoses.^12,26,27^

Large-scale intervention strategies for reducing DWPI abundance in plumbing such as thermal shock^28^ or hyperchlorination^29^ are costly for building managers ($5243 and $11,002 USD annually per 100 water outlets, respectively^28,29^), are often only temporarily effective,^30,31^ and are not suited for small domestic use. An alternative to addressing growth at a building scale would be to reduce DWPIs directly at the point-of-use (POU), which circumvents many of the complexities of addressing DWPI proliferation in the entire distribution system, which can be incorporated into the multibarrier approach to water treatment. POU devices can be more cost-effective than large-scale interventions and quickly employed by concerned consumers or building managers. POU devices can use many mechanisms to treat water (e.g., filtration through media or UV disinfection);^32^ however, the adoption of POU fixtures introduces additional maintenance of these devices, and there is evidence to suggest that colonization may occur in the components themselves. Incorporating silver into POU fixtures is becoming more popular in production and also in healthcare facilities.^33,34^ Silver has been historically used as an antimicrobial agent^35^ and is commonly used in water disinfection in copper–silver ionization systems.^36^ However, the reduction of DWPI abundances is often temporary,^30,37^ and there is concern for the development of antimicrobial resistance after exposure.^38^ When testing the effectiveness of silver-containing POU devices, testing protocols such as ISO 22196:2011, “Measurement of antibacterial activity on plastics and non-porous surfaces”,^39^ are followed, which uses culture-based methods to assess reduction of model organisms in ideal growth conditions. This methodology does not accurately model the complex microbiota and oligotrophic environment of drinking water and may give misleading results due to the induction of the viable but not culturable (VBNC)^40^ state many DWPIs enter when under stress. In fact, independent studies have found that silver-containing fixtures have only minor effects when used on drinking water in real-use scenarios^34,41^ and may unintentionally select for antimicrobial resistance.^42,43^

Silver-containing showerheads that are marketed to reduce microbial loads in shower water are an attractive option for building managers of high-risk facilities (e.g., hospitals or elderly care centers) or concerned consumers looking to reduce the instance of DWPI infection; however, there is little data on how incorporating silver may impact the chemical and microbiological properties of the drinking water. To assess these potential impacts, this study evaluated the transcriptionally active drinking water microbiota from shower water taken from three different silver-containing showerheads (silver-ion-embedded polymer, copper–silver ion, and silver mesh) and were compared to samples from showerheads made with conventional fixture materials.

## Materials and Methods

### Experimental Design

#### Full-Scale Shower Setup and Sampling

Samples analyzed in this study were sourced from the INHALE shower lab at the University of Pittsburgh.^25^ Briefly, this full-scale laboratory consists of three identical shower stalls with three showerheads in each stall, which are separately fed by three hot water heaters and are operated to mimic use patterns of the average adult in the United States by being flushed daily for 8 min (the average showering time) using the average showering temperature for this demographic.^22^ The shower laboratory was conditioned prior to sample collection by initiating daily flushing for one month using showerheads not examined in this study before sampling commenced to ensure there were no prolonged stagnation effects seen in the data. Samples were collected across two sampling campaigns (1—March through May 2021 and 2—February through April 2022). All measurements were performed identically between the campaigns, with the only experimental differences being the dates sampled and the showerheads tested. Both campaigns were conducted over a 12 week period, which is a common manufacturer guideline for antimicrobial showerhead replacement.^44^

#### Showerheads Used

Five different showerheads (four commercially available and one created by the team) were used over the course of this study (Table 1). To simulate conventional shower material use, the two most commonly used showerhead materials (acrylonitrile butadiene styrene (ABS) or oiled bronze) were compared to three antimicrobial silver-containing showerheads, each of which deploys different silver technologies. The commercially available showerheads used in this study are deidentified; however, a brief description of each is provided. From here on, the showerhead referred to as “silver-embedded” was composed of plastic that has silver nanoparticles embedded into the polymer, and the other, referred to from here on as “silver-coated copper mesh”, contained a silver-coated copper mesh component in both the showerhead and hose. The final silver-containing showerhead studied was fabricated by the research team by adding a silver mesh disc (0.356 mm diameter wire, Thermofisher, Waltham, MA) into an ABS plastic showerhead at the junction of the head and hose, which is referred to as “silver mesh”. All showerheads were operated with the same spray pattern and flow rate to ensure any observed differences were due to the presence of silver. Three of each showerhead type was installed (one in each stall) so that triplicate samples from each type could be collected during a sampling event.

**Table 1:** Showerheads Used in This Study

showerhead type	material	commercial availability	sampling campaign^a^
ABS plastic	conventional acrylonitrile butadiene styrene (ABS) polymer	yes	1, 2
metal	conventional oiled bronze	yes	1
silver-embedded	silver nanoparticles incorporated into polymer	yes	1
silver-coated copper mesh	plastic housing with silver-coated copper mesh in both the showerhead and hose	yes	2
silver mesh	ABS plastic housing with silver mesh added	no	2

a Refers to the sampling campaign the showerhead was used in 1: March through May 2021 and 2: February through April 2022.

### Showerhead Materials Testing

Given that the silver component of the silver-embedded showerhead is not visible, we conducted energy-dispersive X-ray spectroscopy (EDS) (Zeiss SIGMA 500VP SEM with Oxford MAX80 EDS detector) to assess its presence in the component of the showerhead described as containing silver. A cross-section was prepared by slicing the unit, enabling analysis of the outer, internal, and inner surfaces (Figure SA2 in the Supporting Information). We evaluated these three locations on cross-sections prepared from two unused showerheads from different manufacturing batches. SEM imaging was performed using an accelerated voltage of 20 kV and a working distance of 10 mm. Samples were coated with ∼2 nm-thick coating of Pd/Au (80%/20%) for conductivity purposes on a Denton sputter coater.

### Sample Collection

Water from each showerhead was sampled biweekly over the course of the 12 week campaign (6 sampling events per campaign, so *n* = 18 samples per showerhead type). Briefly, water sampling entailed collecting the first 1.5 L for each head into a sterile Nalgene bottle. One L was immediately filtered through a 0.2 μm polycarbonate filter (Millipore, Cork, Ireland), and the filter was stored at −80 °C within 20 min of collection to ensure that the maximum amount of RNA was retained prior to extraction, while the remaining water was used for water chemistry analysis and DWPI culturing. A negative control was taken at every sampling event, where deionized water was processed at the same time as sample collection for analysis for all physiochemical and microbiological assessments. Additionally, a filter control was taken at every sampling event where a filter that was used to collect microbial samples was processed to isolate potential contamination from the filter itself.

### Water Quality Measurements

Ten water quality parameters (Table SA1 in the Supporting Information) were measured according to standard methods. Orthophosphate, free chlorine, and total chlorine concentrations were determined at the time of collection using a portable DR900 spectrophotometer (Hach, Loveland, CO, USA). Orthophosphate was determined using the ascorbic acid method,^45^ and free and total chlorine were quantified using the low-range DPD method.^46^ Temperature was monitored on-site using a portable temperature meter (HANNA Instruments, Woonsocket, RI). Oxidation reduction potential and pH were taken at the time of collection using their respective probes (Mettler-Toledo, Columbus, OH). Total and dissolved organic carbon were measured using the Shimadzu TOC-L analyzer using the subtractive method (Shimadzu, Kyoto, Japan). Total and dissolved iron, lead, copper, silver, calcium, and magnesium were determined by using inductively coupled plasma mass spectrometry (PerkinElmer NexION 300 ICP-MS, PerkinElmer, Waltham, MA). Prior to analysis, all dissolved organic carbon and dissolved metal samples were prepared by passing water through a 0.45 μm nylon syringe filter (Thermofisher, Waltham, MA) primed with 5 mL of sample in order to assess the fraction that is more bioavailable. All analyses, except pH and temperature, were performed in triplicate, and the coefficient of variation was at most 12%.

### DWPI Culturing

Culturable DWPI abundances were quantified by the Special Pathogen Laboratory in Pittsburgh, PA. 200 mL water samples were quenched with sodium thiosulfate (enough to quench up to 20 ppm free chlorine in potable water) at the time of collection and subsequently used to culture *Legionella spp*, *Mycobacterium gordonae*, *Mycobacterium mucogenicum*/*phocaicum*, and *P. aeruginosa*. *L. spp*. were quantified using an optimized ISO standard 11,731:2017,^47^*P. aeruginosa* was quantified using a modified ASTM International Standard Test Method D5246,^48^ and NTM were quantified by filter concentrating and decontaminating the sample with 0.2 M KCL-HCL (pH 2.2) prior to plating on Middlebrook 7H10, Mitchison 7H11, and NTM Elite and incubated at 30 °C for 6 weeks.^49^ Only fast-growing NTM (*M. gordonae* and *M. mucogenicum/phocaicum*) were quantified due to the culturing time and competition, with the analytical range of this methodology being 0–600 cfu/mL.

### DWPI Transcriptional Activity from Extracted RNA

RNA from collected water was extracted using the RNeasy Power Water kit (QIAGEN, Hilden, Germany), DNase-treated using the manufacturer’s rigorous treatment protocol of the TURBO DNA-free kit (Invitrogen, Waltham, MA), and then converted to cDNA using the iScript cDNA Synthesis kit (Bio-Rad, Hercules, CA). Between each processing step, the recovery of the genetic material was obtained using the appropriate Qubit assay (Invitrogen, Waltham, MA). The extracted RNA and cDNA were then stored at −80 °C until further analysis. Extracted RNA was assessed using the Agilent 4150 Tapestation (Agilent Technologies) and revealed that median RNA yields and RNA integrity numbers were 6.5 and 0.10 ng/μL, respectively. Reagent controls where molecular-grade water was used instead of the sample were prepared alongside experimental samples to control for potential contamination introduced during processing.

Transcriptional activity (target gene copies/L of shower water) of total bacteria, *L. pneumophila*, *P. aeruginosa*, and NTM was determined using droplet digital polymerase chain reaction (ddPCR) (QX200, Bio-Rad, Hercules, CA) targeting the 16S rRNA gene and taxon-specific genes, respectively (Table SA2 in the Supporting Information). Analysis was conducted in the same manner as described in Pitell and Haig.^25^ Briefly, each 22 μL of ddPCR reaction mixture contained 11 μL of EvaGREEN supermix (Bio-Rad, Hercules, CA), 0.625 mg/mL bovine serum albumin (Invitrogen Corporation, Waltham, MA, USA), 0.2 μM primers (Integrated DNA Technologies, Inc., Coralville, IA), 7.57 μL of water, and 2 μL of the extracted template DNA. Droplets were generated to a 20 μL reaction volume using the automated droplet generation oil for EvaGREEN (Bio-Rad, Hercules, CA), and the plate was heat-sealed. PCR was performed using a C1000 Touch thermal cycler (Bio-Rad Laboratories) within 15 min of droplet generation using the reaction conditions presented in Table SA2 in the Supporting Information. Within 1 h of PCR completion, plates were placed on the droplet reader for quantification. Limits of detection and quantification and thresholds were set for each ddPCR assay (Table SA2 in the Supporting Information), and the transcriptional activity of the target taxa was determined using Quantasoft v1.0.596 following the method described by Lievens et al.^50^ Blanks containing molecular-grade water instead of the template were run on each ddPCR plate as a control.

### 16S rRNA Sequencing

16S rRNA gene amplicon library preparation and sequencing were performed on water samples at Argonne National Laboratory following the Illumina Earth Microbiome Protocol.^51^ Samples were sequenced on an Illumina HiSeq2500 with a total of 8,818,427 raw reads generated. Microbiome analysis was performed using QIIME2 (version 2020.2) with quality filtering performed using the method described in Bolyen et al.^52^ Reads were assigned to operational taxonomic units (OTUs) using a 97% cutoff using the closed reference OTU-picking protocol in QIIME2 (version 2020.2) using the Silva (version 132.5) reference database. The sample data were further processed by identifying and removing all of the OTUs that were ubiquitously present in all negative control samples. All data were processed using the University of Pittsburgh’s Center for Research Computing cluster servers.

### Statistical Analysis

All data was visualized and analyzed using R statistical software (Version 4.0.5). Significant differences (*p*-values < 0.05) in parameters by head type, sample type, and over time were determined using analysis of variance (ANOVA) tests and paired Mann–Whitney U-tests. Functional traits were assigned to the microbial community using PICRUSt2 results using the ggpicrust2 package,^53^ with significance determined by differential abundance analysis conducted with LinDA^54^ with a Benjamini–Hochberg correction. The metabolic predictions generated by PiCRUST2 were deemed reliable since the median nearest sequenced taxon index was 0.058.

Linear mixed-effect models were developed to determine which physiochemical parameters impacted quantities of DWPIs utilizing a stepwise forward and reverse approach to find the model with the lowest Akaike Information Criterion value with a variation inflation factor less than ten.^55^ Power calculations revealed that no more than five explanatory variables should be included in the models, and all DWPI transcriptional activity data was transformed using the BoxCox transformation in R to ensure normal distributions. In addition, all physiochemical data was scaled prior to model generation.

Taxonomic data generated from sequencing were Hellinger transformed prior to analysis to minimize the impact of low abundances of many taxa.^56^ Pairwise dissimilarities between samples were calculated based on the Bray–Curtis dissimilarity index and examined for temporal and spatial patterns in the bacterial community structure by nonmetric multidimensional scaling as implemented in the Vegan package in R.^57^ Significant differences in the microbial community compositions of transcriptionally active microorganisms (Shannon diversity index, Chao’s richness, and Pielou’s evenness) based on showerhead age (days of use since installation of showerheads) and sample type were determined by ANOVA. Relationships between environmental parameters and patterns in transcriptionally active microbial community composition were examined by redundancy analysis (RDA) with significance tested by ANOVA after reducing the overall suite of environmental variables with a stepwise Akaike information criterion model.

## Results and Discussion

### Silver-Containing Showerheads Minimally Impacted Metal Composition in Shower Water

Water quality was assessed over the course of the study. It is important to note that the DW tested met all mandatory regulatory standards upon treatment, so this DW was suitable for direct potable use after treatment by regulatory standards in all cases. While many commonly reported water quality parameters (e.g., pH, free and total chlorine, and organic carbon) were unchanged by showerhead type (Table SA3 in the Supporting Information), the concentrations of many metals were deemed to be significantly different across showerheads, despite minimal differences in absolute concentrations (Table 2). Magnesium, cadmium, and manganese were consistent regardless of the showerhead type (Table 2). Low levels of both cadmium and manganese are often found in DW due to the contribution of natural deposits,^58,59^ and the consistency of these values suggests that the showerhead type does not contribute to these overall concentrations. Stable magnesium concentrations in the water samples could be attributed to the magnesium sacrificial anode rod in the hot water heaters of the INHALE shower laboratory.

**Table 2:** Average ± Standard Deviation of the Total and Dissolved Metal Concentrations (μg/L) Recovered in Shower Water from Different Showerhead Types Across All Sample Timepoints^a^^b^

analyte	average and standard deviation analyte concentration					
	ABS plastic	metal	silver mesh	silver-coated copper mesh	silver-embedded	*p*-value from ANOVA
total silver	3.3 × 10^–3^ ± 0.01	5.6 × 10^–4^ ± 2.4 × 10^–3^	8.9 × 10^–3^ ± 0.02	0.1 ± 0.2	0.01 ± 0.02	**<0.001**
total magnesium	3.9 × 10^3^ ± 625.6	4.0 × 10^3^ ± 664.0	3.9 × 10^3^ ± 791.3	3.9 × 10^3^ ± 802.2	3.9 × 10^3^ ± 636.5	>0.05
total copper	59.1 ± 36.3	86.5 ± 36.1	45.7 ± 22.3	101.3 ± 78.6	88.7 ± 21.8	**<0.001**
total iron	248.8 ± 463.2	440.1 ± 674.7	51.4 ± 14.2	52.7 ± 12.1	417.0 ± 599.5	**0.02**
total lead	0.2 ± 0.3	1.4 ± 1.0	0.3 ± 0.5	0.2 ± 0.2	0.4 ± 0.4	**<0.001**
total zinc	77.9 ± 47.8	121.5 ± 27.4	55.8 ± 58.2	45.2 ± 38.8	118.6 ± 43.8	**<0.001**
total manganese	5.0 ± 13.8	11.4 ± 24.0	0.7 ± 0.8	0.7 ± 0.8	5.7 ± 10.6	>0.05
total cadmium	0.02 ± 0.1	9.0 × 10^–3^ ± 7.4 × 10^–3^	3.3 × 10^–3^ ± 0.02	5.0 × 10^–3^ ± 0.02	4.5 × 10^–3^ ± 7.9 × 10^–3^	>0.05
dissolved silver	0 ± 0.01	0 ± 0	0.02 ± 0.07	0.1 ± 0.1	0.1 ± 0.4	**0.04**
dissolved magnesium	3.8 × 10^3^ ± 608.5	3.9 × 10^3^ ± 609.5	3.9 × 10^3^ ± 700.6	3.9 × 10^3^ ± 740.0	3.8 × 10^3^ ± 583.5	>0.05
dissolved copper	41.9 ± 31.7	63.2 ± 34.8	28.0 ± 14.0	56.5 ± 28.0	67.8 ± 27.0	**<0.001**
dissolved iron	186.0 ± 385.6	320.8 ± 516.5	61.5 ± 50.0	86.7 ± 136.2	307.9 ± 513.2	>0.05
dissolved lead	0.02 ± 0.06	0.6 ± 0.5	0.2 ± 0.7	0 ± 0.06	0.1 ± 0.1	**<0.001**
dissolved zinc	55.2 ± 41.6	99.0 ± 29.7	30.7 ± 30.0	25.4 ± 19.6	91.3 ± 31.9	**<0.001**
dissolved manganese	5.3 ± 22.8	1.7 ± 3.0	0.6 ± 2.0	1.8 ± 5.3	0.6 ± 0.6	>0.05
dissolved cadmium	0 ± 0.01	0.09 ± 0.3	5.6 × 10^–3^ ± 0.02	0.01 ± 0.05	2.8 × 10^–3^ ± 4.6 × 10^–3^	>0.05

a Values that are significantly different (*p* < 0.05) via pairwise assessment between the conventional ABS plastic showerheads and the silver-containing showerheads are italicized

b *n* = 18 for each antimicrobial showerhead and *n* = 36 for ABS showerheads.

Zinc and iron concentrations were significantly lower in the silver mesh and silver-coated copper mesh showerheads compared to the ABS plastic, metal, and silver-embedded showerheads (Table 2). Given that there was no known source of zinc and iron across showerheads, we believe the measured differences are likely due to the distinct sampling events, separated in time. Iron and zinc in DW are associated with pipe corrosion in the DW distribution system; therefore, the decrease in concentrations seen in the latter event (2022) may be linked to the continued formation and subsequent effectiveness of orthophosphate corrosion control scale formation. An orthophosphate corrosion inhibitor was adopted by the city of Pittsburgh in 2019 to reduce lead levels in DW^60^ by creating a protective coating within pipes, which likely also reduced the release of other pipe metals, such as zinc and iron from galvanized steel or other plumbing components, in the water^61^ by the time this study was initiated (spring 2021).

Concentrations of copper and silver were also significantly different by showerhead types (Table 2). This was expected due to the addition of silver to the silver-embedded, silver mesh, and silver-coated copper mesh showerheads and copper in the silver-coated copper mesh showerhead (Table 1). Total copper concentrations were highest in the silver-coated copper mesh showerhead (101.3 μg/L). However, the dissolved copper concentration was only about half of the total and was more consistent in value compared with the other showerheads. Silver also was slightly elevated in water collected from the silver-containing showerheads (8.9 × 10^–3^ μg/L from the silver mesh showerheads, 0.1 μg/L from the silver-coated copper mesh, and 0.01 μg/L from the silver-embedded showerheads) compared to the conventional showerheads (3.3 × 10^–3^ μg/L from the ABS plastic showerheads and 5.6 × 10^–4^ μg/L from the metal showerheads); however, these differences were more likely artifacts of the limit of detection and detection frequency of the analytical method used. Silver was detected more consistently from the silver-containing showerheads (Table 2), with detection frequencies of low concentrations in 100% for silver-coated copper mesh showerheads, 56% for silver mesh showerheads, and 39% for silver-embedded showerheads in the 18 samples taken for each showerhead type. Additionally, silver was detected at low frequency (0% and 8%), often at the limit of quantification in the conventional metal and ABS plastic showerheads, respectively. Typically, silver concentrations in drinking water are below the limit of detection due to the lack of silver in source water and conventional plumbing fixtures, so the low levels and infrequent detection in the conventional fixtures and higher detection from silver-containing showerheads were expected. The lack of consistent silver detection in the samples was unexpected from the silver-containing showerheads since the silver component was hypothesized to contribute to water chemistry by entering the water and providing additional disinfection. Consequently, the silver in these showerheads remains in their components during water use, or it is possible that the silver precipitates into inactive salts within the showerheads.

Silver-Containing Showerheads Did Not Impact Transcriptionally Active DWPI Concentrations in Shower Water

**Figure 1:**
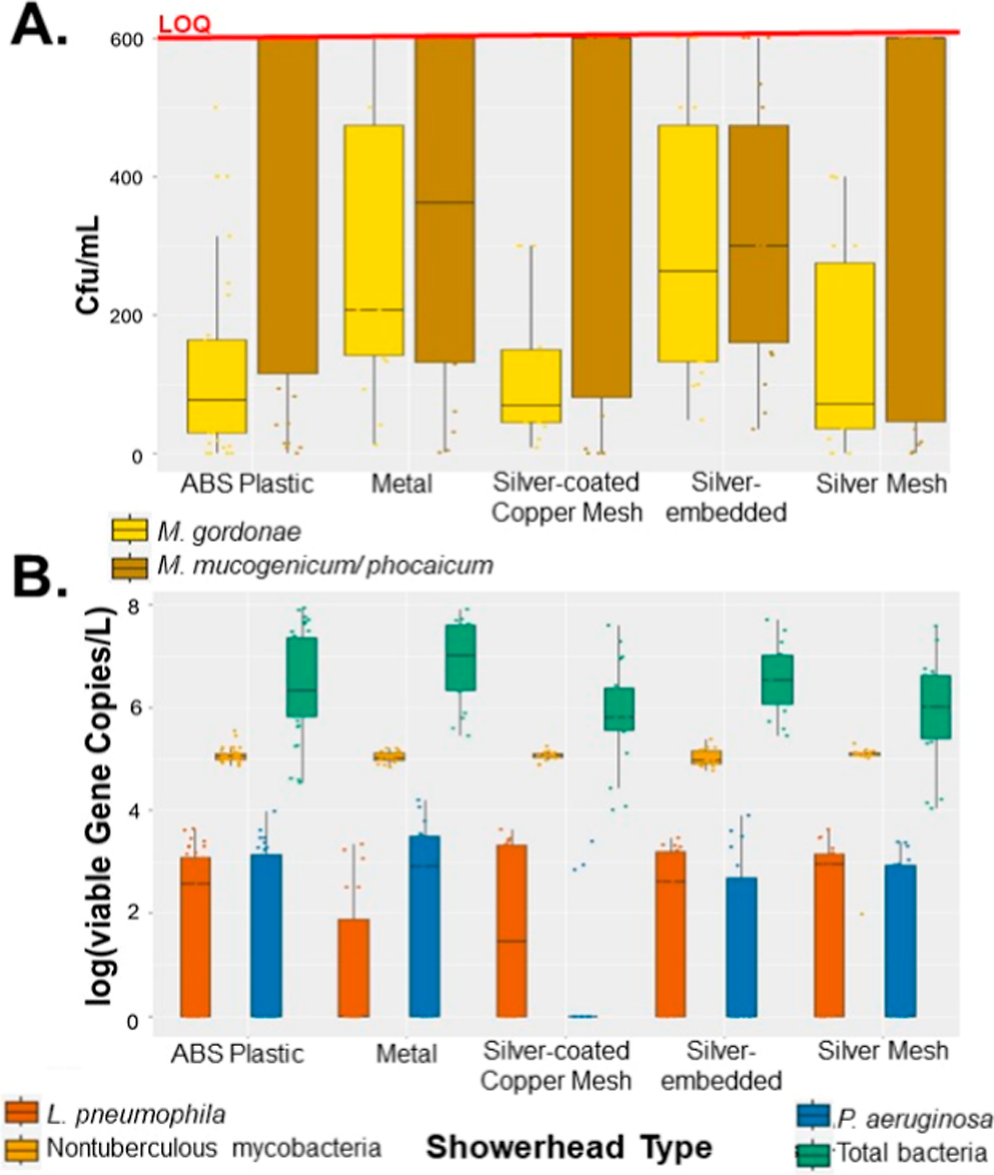
Quantification of DWPIs by showerhead type. (A) Enumeration of *Mycobacterium gordonae* (light yellow) and *Mycobacterium phocacium*/*mucogenicum* (light brown) in culture. The red line labeled “LOQ” refers to the upper limit of quantification of the culturing method. (B) Microbial densities of *Legionella pneumophila* (dark orange), *Pseudomonas aeruginosa* (dark blue), nontuberculous mycobacteria (gold), and total bacteria (green).

This study quantified culturable and transcriptionally active (via RNA) DWPIs. While culturing is used in many studies to determine viable concentrations of DWPIs, it can give an under-representation of microorganisms present due to the ability of DWPIs to enter a viable but not culturable (VBNC) state,^62^ especially when under cellular stress such as in the presence of silver.^41^ Coupling culturing with a culture-independent method, such as ddPCR, allows for VBNC microorganisms to be detected and thus discounts potential differences in colony formation between conventional and silver-containing showerheads. Regardless of methodology, there was no statistically significant difference (*p* > 0.05) between DWPI densities recovered using culture or ddPCR by showerhead type (Figure 1). There were no culture-positive water samples for *L. pneumophila* or *P. aeruginosa*, which was not necessarily surprising given that *L. pneumophila* and *P. aeruginosa* are often transiently detected in hot drinking water systems due to their preference for biofilm growth,^12,63,64^ as well as their ability to enter a VBNC state.^40,62,65^ Specific species of NTM, however, were abundant in cultured water samples sourced from every showerhead type and were too concentrated to quantify via culture in some samples (Figure 1A). The culture results were mostly in agreement with the absolute quantification data (ddPCR).

Transcriptionally active (i.e., transcripts detected by ddPCR of extracted RNA) *L. pneumophila* and *P. aeruginosa* were quantified at low levels in samples from all of the showerheads, whereas NTM were abundant but difficult to recover using the extraction technique due to the unique surface structure of NTM^66^ (Figure 1B). The minor discrepancies in the culturing and molecular data suggest that there are either DWPIs that are in the VBNC state or the microorganisms are not evenly distributed in the water and may not be present at detectable quantities needed for culturing in this model system. Microorganisms can aggregate together and slough from the biofilm forming in the system at any point during water use, where they may have been missed in the representative sample taken (in this case, the first 1.5 L of water).^17^ Unoptimized extraction methodology was likely responsible for the poor NTM RNA recovery that was observed in the ddPCR data, which are notorious for having a thick and waxy cell membrane that resists lysing during extractions.^66^ Further exploration of extraction method development and recovery efficiency calculations for the environmental NTM should be considered to better understand potential discrepancies introduced by the methodology.

The results from culturing and ddPCR analysis suggest that these silver-containing showerheads do not reduce culturable DWPIs or total bacteria or reduce their transcriptional activity in the shower water any more effectively than nonsilver-containing showerheads (*p* > 0.05). Factors such as insufficient silver concentration in the device and subsequent water, silver type in the showerhead, and/or low contact time could contribute to the showerheads’ observed lack of antimicrobial action. For example, the silver-embedded showerhead did not contain any visible metal component, and SEM/EDS analysis found no detectable silver in two separate showerhead components from two distinct manufacturing batches (Figure SA2 in the Supporting Information). It is possible that all the sites on both inserts tested received inconsistent dosing of silver nanoparticles during polymer formation or that there is silver in other components not disclosed by the manufacturer. However, even if silver was present in the device but in a location we could not detect, the device itself resulted in insufficient disinfection. To isolate the effects of pure silver without the potential shortcomings of commercially available showerheads, showerheads containing pure silver mesh were created in-house and tested (termed the “Silver Mesh” showerhead), and even these showerheads did not reduce microbial loads (Figure 1). Because of these findings, it is likely that the standardized testing used to substantiate the marked effectiveness of all the commercially available silver showerheads assessed in this study does not translate into real-world shower applications.

These results are surprising, particularly given that the antimicrobial point-of-use device market is flooded with options that incorporate silver for the purpose of reducing microorganisms in drinking water. Some even provide standardized testing to substantiate the marked effectiveness, including all of the commercially available silver showerheads assessed in this study. Our results importantly elucidate the challenge with translation to real-use conditions. For example, ISO 22196:2011, “Measurement of antibacterial activity on plastics and other non-porous surfaces”, measures the reduction in axenic monoculture after 24 h of direct exposure to the silver-containing material.^39^

Another test, ASTM E 2149 “Standard Test Method for Determining the Antimicrobial Activity of Antimicrobial Agents Under Dynamic Contact Conditions”, measures the reduction in axenic monoculture after 16 h of direct contact with the silver mesh.^67^ Although both methods showed >2 log removal of relevant microorganisms such as *L. pneumophila* and *P. aeruginosa* in standardized testing of the commercially available showerheads used in this study, the methodology itself is a poor proxy to the showering environment. The microbial community in shower water and the biofilms they form have been shown to be complex and intertwined,^68^ oftentimes with functional characteristics in microorganisms such as antimicrobial resistance that vary greatly from laboratory strains.^69^ Reducing these community and water matrix complexities down to pure culture testing in nutrient-rich media may yield results that show the antimicrobial treatment to be more effective than it is for application in shower systems. Another crucial factor to consider is the contact time (amount of antimicrobial material and duration of treatment) used in these standards. Testing the antimicrobial properties over multiple hours may show that it is effective at achieving log removals of microorganisms, but water flowing through the showerhead and hose in application would be exposed to the antimicrobial material for only a much shorter amount of time before exiting the showerhead. If the antimicrobial silver showerheads exhibited poor performance due to insufficient contact time, then ultimately this would translate to no improvement in the microbial water quality. Overall, this study has shown that the standardized methods used to evaluate drinking water antimicrobial materials must consider application to appropriately test their effectiveness and thus generate market claims that are applicable to consumer use.

### Microbial Community Membership Varied between Showerhead Types

Although the antimicrobial silver-containing showerheads did not reduce the transcriptional activity or culturable numbers of DWPIs or total bacteria, there was potential for the presence of silver to affect the greater microbial community. It is known that pipe materials used in drinking water plumbing can affect microorganism growth,^13,14,70,71^ so it is reasonable to consider that the showerheads tested may also influence the microbiome. Silver’s effects on the drinking water microbial community when used as a plumbing material have not been explored to the authors’ knowledge. However, sublethal (low-level) exposure to antimicrobial substances like silver has been shown to select for increased resistance to antimicrobial metals in the microbial community^38,41,42^ in addition to coselecting for antibiotic resistance genes^72^ that may cause unintended public health consequences.

Redundancy analysis revealed that 11.2% of the variance in the microbial community was due to the showerhead type (*p* = < 0.001) (Table SA4 in the Supporting Information). Interestingly, nonmetric multidimensional scaling analysis showed that between the showerhead types, metal showerheads distinctively clustered away from the other showerhead types, and samples taken from the silver-embedded showerhead were also distinct but clustered more loosely (Figure SA3 in the Supporting Information). Additionally, there were statistically significant differences between head types; however, the differences in the absolute magnitude of diversity were fairly small (Figure SA4 in the Supporting Information).

**Figure 2:**
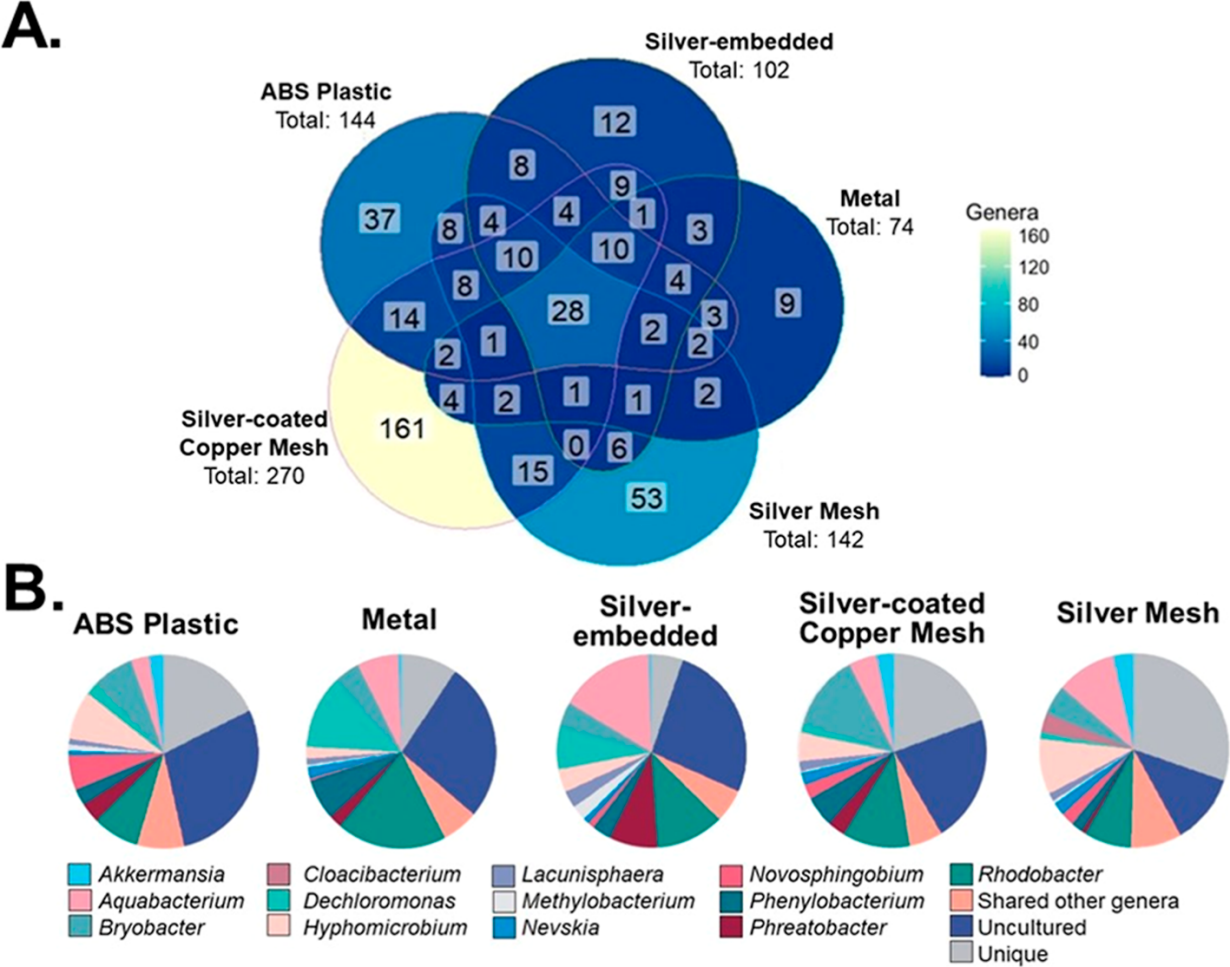
Overlap and proportion of core genera (84% relative abundance on average) in each showerhead type. (A) Venn diagram illustrating the shared and unique genera between different showerhead types and (B) pie charts showing the relative abundance of core genera in each showerhead type. All other shared genera constituting <30% relative abundance each were combined in the “shared other genera” category, and all genera not detected in all showerhead types were combined in the “unique” category.

Analyzing the microbial community composition of transcriptionally active microorganisms revealed more distinct differences among the showerhead types. In terms of genera detected in the different showerhead types, the metal showerhead had the lowest absolute number of total genera (genera = 74, OTU = 121), followed by the silver-embedded (genera = 102, OTU = 203), silver mesh (genera = 142, OTU = 239), ABS plastic (genera = 144, OTU = 293), and the silver-coated copper mesh (genera = 270, OTU = 802) (Figure 2A). Interestingly, two-thirds of the genera detected within silver-containing showerheads were unique (i.e., not shared) with the other showerhead types (Figure 2A). When compared to the conventionally used ABS showerhead, the heterogeneity of the microbial community recovered from the silver-containing showerheads both in the number of genera detected and the minimal overlap in the specific taxa suggests that the actual form of the silver in the showerhead more closely influenced the microbial community transcriptional activity than simply the presence or absence of silver.

Looking at the shared, or core, microbiome identified in all water samples, *Pseudomonadota* was the dominant phylum, accounting for 57% of the core microbiome, with *Alphaproteobacteria* accounting for 58% of the phylum members detected. Microorganisms from *Pseudomonadota* have been reported as the major member of the drinking water microbiome in many studies^68,73−76^ and are known to thrive in chlorinated systems in both the bulk water and biofilms.^68,76^ Further, the phyla contains many of the Gram-negative DWPIs that have been identified to be of public health concern,^1^ as well as being a known reservoir for antimicrobial resistance genes.^73^ Interestingly at the genus level, only 28 genera were shared across all showerhead types (Figure 2B). These 28 included genera associated with DWPIs (e.g., *Legionella* and *Pseudomonas*), which is in agreement with the absolute quantification data where these microorganisms were detected in low quantities, as well as *Burkholderia*–*Caballeronia*–*Paraburkholderia*, another emerging DWPI that was not targeted for direct quantification in this study. The remaining core genera found in this study have been consistently found in high relative abundance in previous DW studies,^77,78^ although interestingly there were differences in the specific relative abundances between the showerheads. For example, the conventional ABS plastic showerhead had higher relative abundances associated with *Novosphingobium* than the other showerheads, which has been observed in other water systems.^79−81^*Dechloromonas* was found in higher abundance in the metal showerheads. This genus was found to dominate a model distribution system constructed of steel, likely due to it supporting various redox reactions.^81^ Given its dominance in metal showerhead samples, it can be assumed that distinct environmental conditions have been formed to favor the proliferation of facultative anaerobes like *Dechloromonas*. Regardless of only 28 core taxa being shared across all samples, these genera accounted for 70–95% of the relative abundance in the samples, suggesting that the significant differences in community composition observed are due to rare taxa. Overall, sequencing analysis results on the water samples in this study corroborated the proposed hypothesis that the materials within the showerheads tested influenced the microbial community even if they do not impact molecular transcription.

### Showerhead Type Impacted Microbiome Functionality

**Figure 3:**
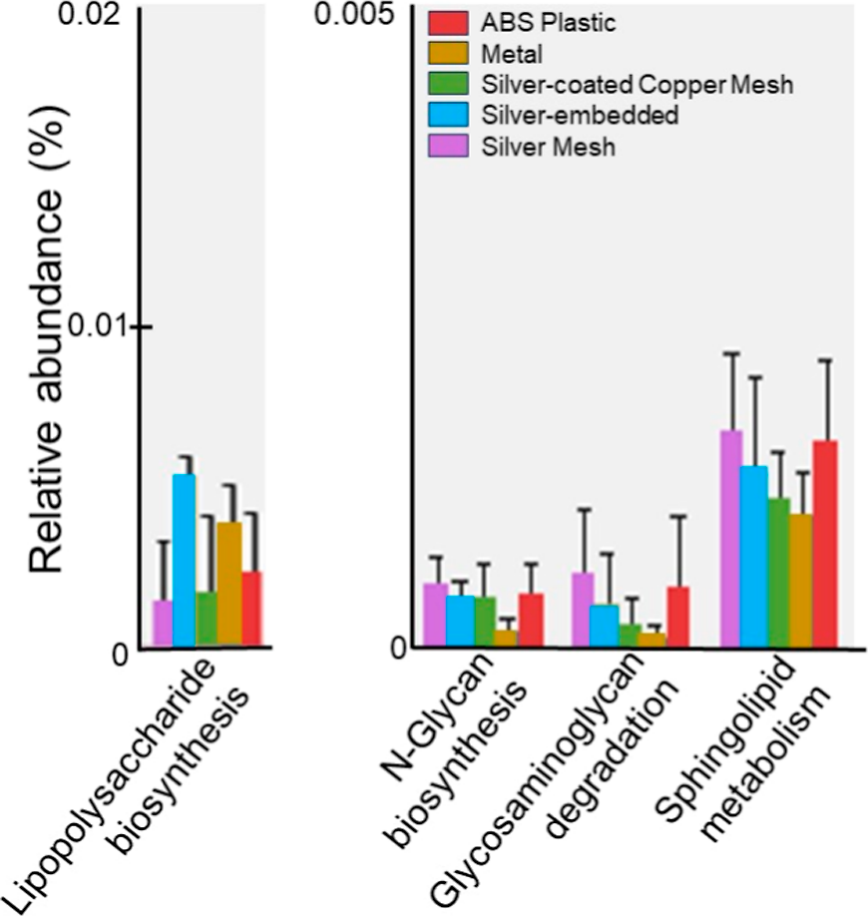
Average relative abundance and standard deviation of statistically significant functional traits in water samples by showerhead type obtained from PICRUSt analysis.

Although >70% of the genera and 52.5% of OTU relative abundances were shared between all showerhead types, there were distinct differences in predicted active community functionality traits (Figure 3). Particularly, important functional differences that impact biofilm development and possible pathogenicity were significantly affected, such as the synthesis of lipopolysaccharides (LPS) and *N*-glycans and the metabolism of glycosaminoglycans and sphingolipids. LPS are known to be essential components of the Gram-negative bacterial cell membranes and can elicit a response in the human immune system.^82^ Although LPS may reduce biofilm-forming capabilities of microorganisms,^83^ its presence indicates higher levels of Gram-negative microorganisms and, as a consequence, greater potential pathogenicity.^84^ Interestingly, the known silver-containing showerheads (the silver-coated copper mesh and silver mesh) had lower relative abundances of LPS synthesis than the conventional showerheads, which suggests that the silver-containing showerheads may select for more Gram-positive organisms and microorganisms that have better biofilm-forming characteristics, and thus, the microbiome from these showerheads may be more prone to form biofilms based on this predictive analysis. This hypothesis is supported by silver mesh showerheads having the highest levels of *N*-glycan biosynthesis and degradation of glycosaminoglycans and sphingolipids (Figure 3)—traits crucial for biofilm formation and stress tolerance.^85,86,86,87^ It is not surprising that microorganisms in direct contact with pure silver would display heightened functional traits for stress management, with the formation of biofilms representing a well-documented microbial strategy for surviving physical, chemical, and biological stress.^88,89^ Although we see differences in predicted functionality with respect to showerhead type, it should be noted that PICRUSt analysis infers functionality based on taxonomy and does not directly interpret gene expression, so these traits in the microbiome are theoretical, and future transcriptomic and proteomic studies are needed to truly assess what functional differences are present. Additionally, little is known about these specific pathways and characteristics in the DW microbiome, so further research must be conducted to fully understand how the increase of these specific traits is functioning within the microbial community.

Though the nonlethal effects of silver on the DW microbiome have not been extensively researched, the work that has been published surrounding the effects of silver has corroborated trends in this data set.^90−96^ The microbial community of water treated with copper–silver ionization has been reported to increase the number of rare taxa in the treated water but did not significantly alter the dominant microbiome.^91^ In addition, silver amendment to stainless steel pipes did not impact biofilm formation but increased the number of rare taxa detected compared to plain stainless steel.^92^ Looking holistically at the use of silver as an antimicrobial in other environmental matrices, its impacts on the microbiome seem to vary based on the state and possible bioavailability of silver used (e.g., nanoparticle or ion^90,93^), what other chemicals are present as either surface stabilizers or anions,^94,96^ and whether the exposure is continuous or an isolated event.^94−96^ Because of this, it is essential for collaboration between engineering and materials science to fully characterize how materials influence the microbial community in regards to optimal showerhead material choice for additional water disinfection or to optimize disinfection strategies using silver and other intrinsic antimicrobials by designing showerheads with effective contact times.

### Showerhead Age Influenced the Microbiota

The showerhead age, or the amount of time of daily use of the showerhead, was the second most important parameter that was identified in the redundancy analysis and the permutational multivariate analysis of variance on the Bray–Curtis distances (*p* = > 0.001) (Table SA4 in the Supporting Information). Plumbing age has been identified to influence the microbiome due to material degradation and subsequent release into water as well as the opportunity to allow biofilms to develop,^14,97−99^ which is expected to influence the microbiome of the resulting shower water due to the dynamic attachment and detachment of the biofilm.^17^ While the effect of the plumbing age of fixtures on the biofilm has not been extensively explored, there is evidence to suggest that biofilms undergo marked changes in behavior after initial colonization (around 30 days^18^), which then influences the planktonic microbiota in the shower water that can reach consumers when showering.^18,25^ Despite the lack of characterization of biofilm buildup, manufacturers of the silver-embedded showerhead dictate to consumers to change the silver-containing insert and to conduct a chemical cleaning treatment on the showerheads after 12 weeks of use.^44^ Biofilms are known to be reservoirs for DWPIs^12,27,63,70,92^ and other microorganisms, so determining how the biofilm formation stages impact the microbial water quality is essential for understanding how showerhead age influences potential consumer exposure.

**Figure 4:**
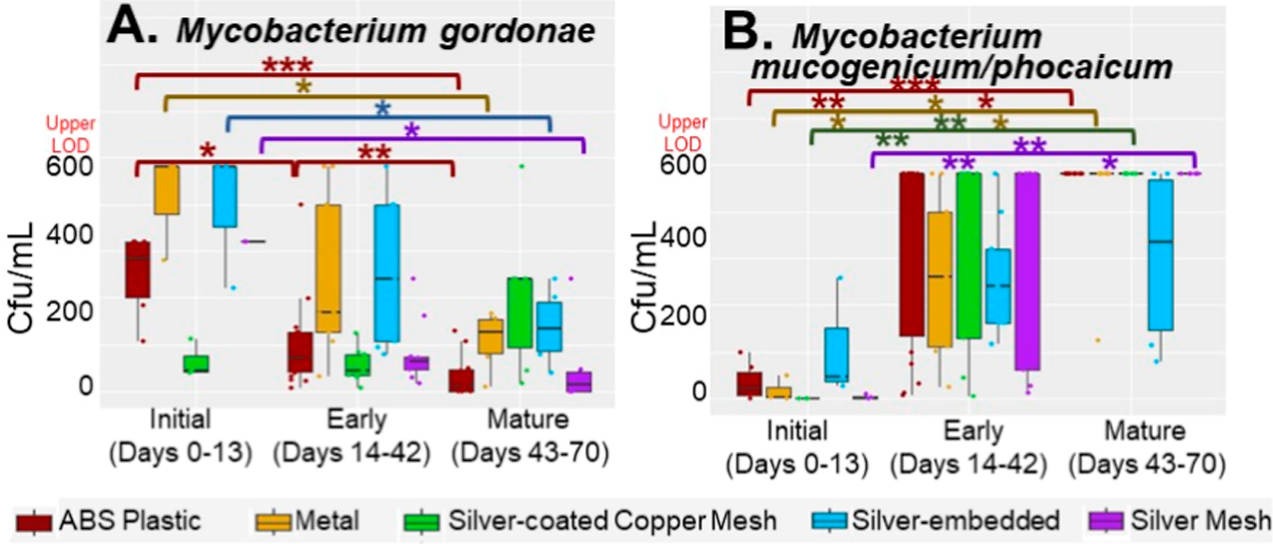
Culturable concentrations of (A) *Mycobacterium gordonae* and (B) *Mycobacterium mucogenicum*/*phocaicum* from ABS plastic (red), metal (yellow), silver-coated copper mesh (green), silver-embedded (blue), and silver mesh (purple) showerheads by the biofilm formation stage. Significant differences at *p*-values <0.05, <0.01, and <0.001 are denoted by *, **, and ***, respectively, in the corresponding color of the showerhead type (e.g., the red bracket and asterisks correspond to differences in the ABS showerhead). The upper limit of quantification for this method was 600 cfu/mL.

There was little variation in the transcriptional activity of DWPIs recovered from each showerhead type with respect to showerhead age (Figure SA5 in Supporting Information); however, the abundances of individual species of NTM in culture were significantly affected (Figure 4). *Culturable M*. *gordonae*, a slow-growing NTM species^100^ known to be ubiquitous and abundant in drinking water,^101^ significantly decreased in concentration as the biofilm developed (Figure 4A) in the ABS plastic, metal, silver-embedded, and silver mesh showerheads (*p* < 0.05). This decreasing trend over time has been previously observed in an investigation of a pseudo-outbreak of *M. gordonae* that linked the new plumbing of a hospital wing to increased water densities and clinical incidence.^102^ Interestingly, this decreasing trend was not observed in the silver-coated copper mesh showerhead samples, where *culturable M*. *gordonae* concentrations were consistent at all time points, resembling concentrations observed during the mature biofilm phase seen in the other showerhead types. This suggests that the presence of copper within a showerhead may reduce *M. gordonae* densities from shower water; however, this connection needs to be further explored. Interestingly, the inverse relationship between showerhead age and culturable NTM was observed for the faster-growing^103^*M. mucogenicum*/*phocacium* (Figure 4B): as showerhead age increases, culturable *M. mucogenicum*/*phocacium* densities significantly increase for the samples taken from the ABS plastic, metal, silver-coated copper mesh, and silver mesh showerheads (*p* = < 0.05). This positive association was also apparent in the silver-embedded showerhead samples but was not statistically significant (*p* > 0.05). This trend is more aligned with conventional drinking water biofilm theory, where the biofilm functions as a reservoir for microorganisms and contributes to the bulk water densities during sloughing events.

These trends in NTM were also observed in a study conducted by Yang et al.^104^ in established DW distribution systems and building plumbing, where *M. gordonae* was more abundant in biofilm swab samples and *M. mucogenicum* was more abundant in water samples.^104^ It is known that *Mycobacterium spp*. can be cooperative across species in clinical infection,^105^ and many genera are often present at the same time in environmental samples,^106^ so it is possible that *M. gordonae* did not assimilate to the biofilm until after weeks of continuous use and, consequently, was found in the water fraction in early weeks. *M. mucogenicum*/*phocaicum*, on the other hand, may have established biofilm growth earlier due to their faster growth rate, then thus experienced accelerated biofilm dynamics so that sloughing into the bulk water could occur later, explaining the higher densities in the later sampling events. Further work must be conducted to explore the role of biofilm formation and how it can influence planktonic concentrations of DWPIs in resulting shower water.

## Conclusions

The results of the work outlined in this paper demonstrated that silver-containing showerheads did not produce significantly different DWPI transcriptional activity over the duration of 12 weeks but impacted the abundances of metals based on the showerhead type (despite the scale of these differences being small). Although the quantities of culturable or molecularly detected DWPIs remained unchanged, the incorporation of silver, along with the specific type of silver utilized in the showerhead, influenced the broader microbial community and promoted selection of biofilm-specific functional traits. These findings suggest that material type affects the drinking water microbiome beyond marketed antimicrobial properties, and more work must be done on a mechanistic level to characterize the effects of silver in these exposure conditions. Additionally, this work highlighted showerhead age as an important parameter in explaining microbial trends for NTM in shower water, which may be tied to the biofilm formation in the showerheads and hoses. Because of the impacts of age on *M. ycobacterium* spp. seen in this study in particular, showerhead and plumbing age should be considered when doing future quantitative microbial risk assessment. The lack of culturable *L. pneumophila* and *P. aeruginosa* recovered from this model plumbing system, although representative of a domestic system, highlights an avenue for additional studies evaluating silver-containing showerheads in systems that have been intentionally inoculated with larger quantities of these microorganisms. It should be stressed that molecularly detected DWPI transcripts in this study are lower than have been seen in other studies; however, culturable amounts of NTM were on par with other studies. It is possible that removal performances of the shower heads would be different if concentrations were higher; therefore, future studies could consider inoculating plumbing systems with DWPIs to evaluate showerhead performance at higher microbial loads. However, much caution should be applied in any such spiking studies to avoid creating artificial environments which do not reflect the microbiome and DWPI concentrations naturally found in building water. This work has highlighted the need for rigorous engineering and testing under real-use conditions of antimicrobial water fixtures and has opened the doors for continued innovation to technologies designed to reduce DWPIs.

## Data Availability

The environmental and sequencing data that support the findings of this study are openly available in Zenodo at https://zenodo.org/uploads/10976012, reference number 10.5281/zenodo.10976012.

## Supporting Information Available

Table SA1: Different physical and chemical water quality parameters measured in this study and corresponding analytical method used. Table SA2: Molecular primers, thresholds, and assay sensitivity for ddPCR analysis. Table SA3: Average values of measured water chemistry parameters. Table SA4: Summary of generated linear models in water samples. In the model components column, ± indicates positive or negative association and the percent of the variance explained by each variable is superscripted. Table SA5: Average diversity values of water samples ± standard deviation. Sample size is denoted in parentheses under value. Figure SA1: Schematic of full-scale shower laboratory set-up. The blue X indicates the aerosol sampling port in the Plexiglass door, the black X indicates where the copper pipe from the hot water heater connects to the shower stall, and the red X’s correspond to each showerhead position. Figure SA2: Summary of SEM–EDS analysis conducted on silver-containing inserts of the silver-embedded head. (A) Annotated diagram of the sampling sites of the shower insert, with A1 being the “top” of the insert, A2 being taken from the side wall, and A3 being the “bottom” of the insert. (B) Representative EDS scan from an insert with red corresponding to sampling site A1, blue corresponding to sampling site A2, and yellow corresponding to sampling site A3. (C) Representative SEM image taken from the showerhead insert, with maps of (i) oxygen and (ii) titanium. Figure SA3: Non-metric multidimensional scaling plot of water samples by showerhead type. Figure SA4: Shannon diversity calculated from water samples by showerhead type. Figure SA5: Viable concentrations of (A) *L. pneumophila*, (B) *P. aeruginosa*, (C) *M. gordonae*, (D) NTM, (E) total bacteria, and (F) *M. mucogenicum*/*phocaicum* for ABS plastic (red), metal (yellow), silver-coated copper mesh (green), silver-embedded (blue), and silver mesh (purple) showerheads by biofilm formation stage. Significant differences are marked with a colored bracket and star corresponding to the showerhead type and *p*-value (* = *p* ≤ 0.05, ** = *p* ≤ 0.01, *** = *p* ≤ 0.001) (PDF)

## References

[ref1] Proctor C.; Garner E.; Hamilton K. A.; Ashbolt N. J.; Caverly L. J.; Falkinham J. O.; Haas C. N.; Prevost M.; Prevots D. R.; Pruden A.; Raskin L.; Stout J.; Haig S.-J. Tenets of a Holistic Approach to Drinking Water-Associated Pathogen Research, Management, and Communication. Water Res. 2022, 211, 117997 10.1016/j.watres.2021.117997. 34999316 PMC8821414

[ref2] Collier S. A.; Deng L.; Adam E. A.; Benedict K. M.; Beshearse E. M.; Blackstock A. J.; Bruce B. B.; Derado G.; Edens C.; Fullerton K. E.; Gargano J. W.; Geissler A. L.; Hall A. J.; Havelaar A. H.; Hill V. R.; Hoekstra R. M.; Reddy S. C.; Scallan E.; Stokes E. K.; Yoder J. S.; Beach M. J. Estimate of Burden and Direct Healthcare Cost of Infectious Waterborne Disease in the United States. Emerg Infect Dis 2021, 27, 140–149. 10.3201/eid2701.190676. 33350905 PMC7774540

[ref3] Findings | Water-Related Topics | Healthy Water; CDC. https://www.cdc.gov/healthywater/surveillance/burden/findings.html (accessed 2023–08–07).

[ref4] Adjemian J.; Olivier K. N.; Seitz A. E.; Falkinham J. O.; Holland S. M.; Prevots D. R. Spatial Clusters of Nontuberculous Mycobacterial Lung Disease in the United States. Am. J. Respir. Crit. Care Med. 2012, 186 (6), 553–558. 10.1164/rccm.201205-0913OC. 22773732 PMC3480533

[ref5] Surveillance for Waterborne Disease Outbreaks Associated with Drinking Water and Other Nonrecreational Water — United States 2009–2010. https://www.cdc.gov/mmwr/preview/mmwrhtml/mm6235a3.htm (accessed 2022–10–21). PMC4585624 24005226

[ref6] Cunha B. A.; Burillo A.; Bouza E. Legionnaires’ Disease. Lancet 2016, 387 (10016), 376–385. 10.1016/S0140-6736(15)60078-2. 26231463

[ref7] Mather M.; Jacobsen L. A.; Ard K. M. P.. Www.Prb.Org. Population Bulletin. Popul. Bull. 2015.

[ref8] Patel M.; Chen J.; Kim S.; Garg S.; Flannery B.; Haddadin Z.; Rankin D.; Halasa N.; Talbot H. K.; Reed C. Analysis of MarketScan Data for Immunosuppressive Conditions and Hospitalizations for Acute Respiratory Illness, United States. Emerg Infect Dis 2020, 26, 1720–1730. 10.3201/eid2608.191493. 32348234 PMC7392442

[ref9] Pseudomonas aeruginosa Infection | HAI; CDC. https://www.cdc.gov/hai/organisms/pseudomonas.html (accessed 2022–11–30).

[ref10] Baldwin S. L.; Larsen S. E.; Ordway D.; Cassell G.; Coler R. N. The Complexities and Challenges of Preventing and Treating Nontuberculous Mycobacterial Diseases. PLoS Negl. Trop. Dis. 2019, 13 (2), e0007083 10.1371/journal.pntd.0007083. 30763316 PMC6375572

[ref11] Collins S.; Stevenson D.; Bennett A.; Walker J. Occurrence of Legionella in UK Household Showers. Int. J. Hyg. Environ. Health 2017, 220 (2), 401–406. 10.1016/j.ijheh.2016.12.001. 27964907

[ref12] Feazel L. M.; Baumgartner L. K.; Peterson K. L.; Frank D. N.; Harris J. K.; Pace N. R. Opportunistic Pathogens Enriched in Showerhead Biofilms. Proc. Natl. Acad. Sci. U.S.A. 2009, 106 (38), 16393–16399. 10.1073/pnas.0908446106. 19805310 PMC2752528

[ref13] Proctor C. R.; Dai D.; Edwards M. A.; Pruden A. Interactive Effects of Temperature, Organic Carbon, and Pipe Material on Microbiota Composition and Legionella Pneumophila in Hot Water Plumbing Systems. Microbiome 2017, 5 (1), 130 10.1186/s40168-017-0348-5. 28978350 PMC5628487

[ref14] Cullom A. C.; Martin R. L.; Song Y.; Williams K.; Williams A.; Pruden A.; Edwards M. A. Critical Review: Propensity of Premise Plumbing Pipe Materials to Enhance or Diminish Growth of Legionella and Other Opportunistic Pathogens. Pathogens 2020, 9 (11), 957–1034. 10.3390/pathogens9110957. 33212943 PMC7698398

[ref15] Proctor R.; C; Gächter M.; Kötzsch S.; Rölli F.; Sigrist R.; Walser J.-C.; Hammes F. Biofilms in Shower Hoses – Choice of Pipe Material Influences Bacterial Growth and Communities. Environ. Sci.:Water Res. Technol. 2016, 2 (4), 670–682. 10.1039/C6EW00016A.

[ref16] Berry D.; Xi C.; Lutgarde R. Microbial Ecology of Drinking Water Distribution s: Full Text Finder Results. Curr. Opin. Biotechnol. 2006, 17 (3), 297–302. 10.1016/j.copbio.2006.05.007. 16701992

[ref17] Costerton J. W.; Lewandowski Z.; Caldwell D. E.; Korber D. R.; Lappin-Scott H. M. Microbial Biofilms. Annu. Rev. Microbiol. 1995, 49 (1), 711–745. 10.1146/annurev.mi.49.100195.003431. 8561477

[ref18] Morvay A. A.; Decun M.; Scurtu M.; Sala C.; Morar A.; Sarandan M. Biofilm Formation on Materials Commonly Used in Household Drinking Water Systems. Water Sci. Technol.: Water Supply 2011, 11 (2), 252–257. 10.2166/ws.2011.053.

[ref19] Thomson R.; Tolson C.; Carter R.; Coulter C.; Huygens F.; Hargreaves M. Isolation of Nontuberculous Mycobacteria (NTM) from Household Water and Shower Aerosols in Patients with Pulmonary Disease Caused by NTM. J. Clin. Microbiol. 2013, 51 (9), 3006–3011. 10.1128/JCM.00899-13. 23843489 PMC3754680

[ref20] Falkinham J. O.; Iseman M. D.; de Haas P.; van Soolingen D. Mycobacterium Avium in a Shower Linked to Pulmonary Disease. J. Water Health 2008, 6 (2), 209–213. 10.2166/wh.2008.232. 18209283

[ref21] Saving water in your home | Portland.gov. https://www.portland.gov/water/water-efficiency-programs/save-water-home (accessed 2022–11–23).

[ref22] Wilkes C. R.; Mason A. D.; Hern S. C. Probability Distributions for Showering and Bathing Water-Use Behavior for Various U.S. Subpopulations. Risk Anal 2005, 25 (2), 317–337. 10.1111/j.1539-6924.2005.00592.x. 15876207

[ref23] Nazaroff W. W. Indoor Bioaerosol Dynamics. Indoor Air 2016, 26 (1), 61–78. 10.1111/ina.12174. 25483392 PMC7165847

[ref24] Zhou Y.; Mui K. W.; Wong L. T.; Tsui P. H.; Chan W. K. Aerosol Generation Rates for Showerheads. Build. Serv. Eng. Res. Technol. 2019, 40 (5), 595–610. 10.1177/0143624418824839.

[ref25] Pitell S.; Haig S.-J. Assessing the Impact of Anti-Microbial Showerheads on the Prevalence and Abundance of Opportunistic Pathogens in Shower Water and Shower Water-Associated Aerosols. Front. Microbiomes 2023, 2. 10.3389/frmbi.2023.1292571. PMC12993570 41853348

[ref26] Proctor C. R.; Reimann M.; Vriens B.; Hammes F. Biofilms in Shower Hoses. Water Res. 2018, 131, 274–286. 10.1016/j.watres.2017.12.027. 29304381

[ref27] Gebert M. J.; Delgado-Baquerizo M.; Oliverio A. M.; Webster T. M.; Nichols L. M.; Honda J. R.; Chan E. D.; Adjemian J.; Dunn R. R.; Fierer N. Ecological Analyses of Mycobacteria in Showerhead Biofilms and Their Relevance to Human Health. mBio 2018, 9 (5), e01614 10.1128/mBio.01614-18. 30377276 PMC6212831

[ref28] Bédard E.; Boppe I.; Kouamé S.; Martin P.; Pinsonneault L.; Valiquette L.; Racine J.; Prévost M. Combination of Heat Shock and Enhanced Thermal Regime to Control the Growth of a Persistent Legionella Pneumophila Strain. Pathogens 2016, 5 (2), 35 10.3390/pathogens5020035. 27092528 PMC4931386

[ref29] Orsi G. B.; Vitali M.; Marinelli L.; Ciorba V.; Tufi D.; Del Cimmuto A.; Ursillo P.; Fabiani M.; De Santis S.; Protano C.; Marzuillo C.; De Giusti M. Legionella Control in the Water System of Antiquated Hospital Buildings by Shock and Continuous Hyperchlorination: 5 Years Experience. BMC Infect. Dis. 2014, 14 (1), 394 10.1186/1471-2334-14-394. 25027499 PMC4223580

[ref30] Stout J. E.; Lin Y.-S. E.; Goetz A. M.; Muder R. R. Controlling Legionella in Hospital Water Systems: Experience with the Superheat-and-Flush Method and Copper-Silver Ionization. Infect. Control Hosp. Epidemiol. 1998, 19 (12), 911–914. 10.2307/30142016. 9872527

[ref31] Marchesi I.; Marchegiano P.; Bargellini A.; Cencetti S.; Frezza G.; Miselli M.; Borella P. Effectiveness of Different Methods to Control Legionella in the Water Supply: Ten-Year Experience in an Italian University Hospital. J. Hosp. Infect. 2011, 77 (1), 47–51. 10.1016/j.jhin.2010.09.012. 21131100

[ref32] Wu J.; Cao M.; Tong D.; Finkelstein Z.; Hoek E. M. V. A Critical Review of Point-of-Use Drinking Water Treatment in the United States. Npj Clean Water 2021, 4 (1), 40 10.1038/s41545-021-00128-z.

[ref33] Parkinson J.; Baron J. L.; Hall B.; Bos H.; Racine P.; Wagener M. M.; Stout J. E. Point-of-Use Filters for Prevention of Health Care–Acquired Legionnaires’ Disease: Field Evaluation of a New Filter Product and Literature Review. Am. J. Infect. Control 2020, 48 (2), 132–138. 10.1016/j.ajic.2019.09.006. 31668765

[ref34] Reasoner D. J.; Blannon J. C.; Geldreich E. E. Microbiological Characteristics of Third-Faucet Point-of-Use Devices. J. AWWA 1987, 79 (10), 60–66. 10.1002/j.1551-8833.1987.tb02925.x.

[ref35] Barillo D. J.; Marx D. E. Silver in Medicine: A Brief History BC 335 to Present. Burns 2014, 40, S3–S8. 10.1016/j.burns.2014.09.009. 25418435

[ref36] Liu Z.; Stout J. E.; Tedesco L.; Boldin M.; Hwang C.; Diven W. F.; Yu V. L. Controlled Evaluation of Copper-Silver Ionization in Eradicating Legionella Pneumophila from a Hospital Water Distribution System. J. Infect. Dis. 1994, 169 (4), 919–922. 10.1093/infdis/169.4.919. 8133111

[ref37] Rohr U.; Senger M.; Selenka F.; Turley R.; Wilhelm M. Four Years of Experience with Silver-Copper Ionization for Control of Legionella in a German University Hospital Hot Water Plumbing System. Clin. Infect. Dis. 1999, 29 (6), 1507–1511. 10.1086/313512. 10585804

[ref38] Graves J. L.; Tajkarimi M.; Cunningham Q.; Campbell A.; Nonga H.; Harrison S. H.; Barrick J. E. Rapid Evolution of Silver Nanoparticle Resistance in Escherichia Coli. Front. Genet. 2015, 6, 42 10.3389/fgene.2015.00042. 25741363 PMC4330922

[ref39] In2itive. ISO 22196:2011. Viroxy. https://www.viroxylabs.com/microbiological-testing-services/disinfectant-efficacy-testing/iso-221962011/(accessed 2022-08-16).

[ref40] Li L.; Mendis N.; Trigui H.; Oliver J. D.; Faucher S. P. The Importance of the Viable but Non-Culturable State in Human Bacterial Pathogens. Front. Microbiol. 2014, 5, 258 10.3389/fmicb.2014.00258. 24917854 PMC4040921

[ref41] June S. G.; Dziewulski D. M. Copper and Silver Biocidal Mechanisms, Resistance Strategies, and Efficacy for Legionella Control. J. AWWA 2018, 110 (12), E13–E35. 10.1002/awwa.1144.

[ref42] Rodgers M. R.; Blackstone B. J.; Reyes A. L.; Covert T. C. Colonisation of Point of Use Water Filters by Silver Resistant Non-Tuberculous Mycobacteria. J. Clin. Pathol. 1999, 52 (8), 629 10.1136/jcp.52.8.629a. PMC500959 10645237

[ref43] Yetiş O. ¨.; Ali S.; Karia K.; Bassett P.; Wilson P. Persistent Colonisation of Antimicrobial Silver-Impregnated Shower Heads and Hoses Presents A Risk for Acquisition of Pseudomonas Aeruginosa in Healthcare Settings; preprint. Review 2022, 8, 118 10.21203/rs.3.rs-1403389/v1.

[ref44] The Efficacy of the Medi-Shower Silver Impregnated Showerhead. Medi-shower. https://medi-shower.co.uk/studies/the-efficacy-of-the-medishower-silver-impregnated-showerhead (accessed-2021-04-06).

[ref45] Hach USA. Phosphorus, Reactive (Orthophosphate) USEPA PhosVer 3 (Ascorbic Acid) Method, 2022.file:///C:/Users/sarah/Downloads/DOC316.53.01119.pdf.

[ref46] Hach USA. Chlorine, Free and Total, Low Range USEPA DPD Method 2022. file:///C:/Users/sarah/Downloads/DOC316.53.01450_5ed.pdf

[ref47] 14:00–17:00. ISO 11731:2017. ISO. https://www.iso.org/standard/61782.html (accessed 2023-05-10).

[ref48] Standard Test Method for Isolation and Enumeration of Pseudomonas aeruginosa from Water. https://www.astm.org/d5246-19.html (accessed 2023–05–10).

[ref49] Alexander K. J.; Furlong J. L.; Baron J. L.; Rihs J. D.; Stephenson D.; Perry J. D.; Stout J. E. Evaluation of a New Culture Medium for Isolation of Nontuberculous Mycobacteria from Environmental Water Samples. PLoS One 2021, 16 (3), e0247166 10.1371/journal.pone.0247166. 33657154 PMC7928522

[ref50] Lievens A.; Jacchia S.; Kagkli D.; Savini C.; Querci M. Measuring Digital PCR Quality: Performance Parameters and Their Optimization. PLoS One 2016, 11 (5), e0153317 10.1371/journal.pone.0153317. 27149415 PMC4858304

[ref51] Caporaso J. G.; Lauber C. L.; Walters W. A.; Berg-Lyons D.; Huntley J.; Fierer N.; Owens S. M.; Betley J.; Fraser L.; Bauer M.; Gormley N.; Gilbert J. A.; Smith G.; Knight R. Ultra-High-Throughput Microbial Community Analysis on the Illumina HiSeq and MiSeq Platforms. ISME J. 2012, 6 (8), 1621–1624. 10.1038/ismej.2012.8. 22402401 PMC3400413

[ref52] Bolyen E.; Rideout J. R.; Dillon M. R.; Bokulich N. A.; Abnet C. C.; Al-Ghalith G. A.; Alexander H.; Alm E. J.; Arumugam M.; Asnicar F.; Bai Y.; Bisanz J. E.; Bittinger K.; Brejnrod A.; Brislawn C. J.; Brown C. T.; Callahan B. J.; Caraballo-Rodríguez A. M.; Chase J.; Cope E. K.; Da Silva R.; Diener C.; Dorrestein P. C.; Douglas G. M.; Durall D. M.; Duvallet C.; Edwardson C. F.; Ernst M.; Estaki M.; Fouquier J.; Gauglitz J. M.; Gibbons S. M.; Gibson D. L.; Gonzalez A.; Gorlick K.; Guo J.; Hillmann B.; Holmes S.; Holste H.; Huttenhower C.; Huttley G. A.; Janssen S.; Jarmusch A. K.; Jiang L.; Kaehler B. D.; Kang K. B.; Keefe C. R.; Keim P.; Kelley S. T.; Knights D.; Koester I.; Kosciolek T.; Kreps J.; Langille M. G. I.; Lee J.; Ley R.; Liu Y.-X.; Loftfield E.; Lozupone C.; Maher M.; Marotz C.; Martin B. D.; McDonald D.; McIver L. J.; Melnik A. V.; Metcalf J. L.; Morgan S. C.; Morton J. T.; Naimey A. T.; Navas-Molina J. A.; Nothias L. F.; Orchanian S. B.; Pearson T.; Peoples S. L.; Petras D.; Preuss M. L.; Pruesse E.; Rasmussen L. B.; Rivers A.; Robeson M. S.; Rosenthal P.; Segata N.; Shaffer M.; Shiffer A.; Sinha R.; Song S. J.; Spear J. R.; Swafford A. D.; Thompson L. R.; Torres P. J.; Trinh P.; Tripathi A.; Turnbaugh P. J.; Ul-Hasan S.; van der Hooft J. J. J.; Vargas F.; Vázquez-Baeza Y.; Vogtmann E.; von Hippel M.; Walters W.; Wan Y.; Wang M.; Warren J.; Weber K. C.; Williamson C. H. D.; Willis A. D.; Xu Z. Z.; Zaneveld J. R.; Zhang Y.; Zhu Q.; Knight R.; Caporaso J. G. Reproducible, Interactive, Scalable and Extensible Microbiome Data Science Using QIIME 2. Nat. Biotechnol. 2019, 37 (8), 852–857. 10.1038/s41587-019-0209-9. 31341288 PMC7015180

[ref53] Yang C.; Mai J.; Cao X.; Burberry A.; Cominelli F.; Zhang L. Ggpicrust2: An R Package for PICRUSt2 Predicted Functional Profile Analysis and Visualization. Bioinformatics 2023, 39, btad470 10.1093/bioinformatics/btad470. 37527009 PMC10425198

[ref54] Zhou H.; He K.; Chen J.; Zhang X. LinDA: Linear Models for Differential Abundance Analysis of Microbiome Compositional Data. Genome Biol. 2022, 23 (1), 95 10.1186/s13059-022-02655-5. 35421994 PMC9012043

[ref55] Haig S.-J.; Kotlarz N.; Kalikin L. M.; Chen T.; Guikema S.; LiPuma J. J.; Raskin L. Emerging Investigator Series: Bacterial Opportunistic Pathogen Gene Markers in Municipal Drinking Water Are Associated with Distribution System and Household Plumbing Characteristics. Environ. Sci.:Water Res. Technol. 2020, 6 (11), 3032–3043. 10.1039/D0EW00723D.

[ref56] Legendre P.; Gallagher E. D. Ecologically Meaningful Transformations for Ordination of Species Data. Oecologia 2001, 129 (2), 271–280. 10.1007/s004420100716. 28547606

[ref57] Oksanen J.; Simpson G. L.; Blanchet F. G.; Kindt R.; Legendre P.; Minchin P. R.; O’Hara R. B.; Solymos P.; Stevens M. H. H.; Szoecs E.; Wagner H.; Barbour M.; Bedward M.; Bolker B.; Borcard D.; Carvalho G.; Chirico M.; Caceres M. D.; Durand S.; Evangelista H. B. A.; FitzJohn R.; Friendly M.; Furneaux B.; Hannigan G.; Hill M. O.; Lahti L.; McGlinn D.; Ouellette M.-H.; Cunha E. R.; Smith T.; Stier A.; Braak C. J. F. T.; Weedon J. Vegan: Community Ecology Package, 2022. https://CRAN.R-project.org/package=vegan (accessed 2023–03-21).

[ref58] Us Epa O. National Primary Drinking Water Regulations. https://www.epa.gov/ground-water-and-drinking-water/national-primary-drinking-water-regulations (accessed 2024–03–24).

[ref59] Us Epa O. Secondary Drinking Water Standards: Guidance for Nuisance Chemicals. https://www.epa.gov/sdwa/secondary-drinking-water-standards-guidance-nuisance-chemicals (accessed 2024–03–24).

[ref60] Spencer-Williams I.; Meyer M.; DePas W.; Elliott E.; Haig S.-J. Assessing the Impacts of Lead Corrosion Control on the Microbial Ecology and Abundance of Drinking-Water-Associated Pathogens in a Full-Scale Drinking Water Distribution System. Environ. Sci. Technol. 2023, 57 (48), 20360–20369. 10.1021/acs.est.3c05272. 37970641 PMC10702490

[ref61] Clark B. N.; Masters S. V.; Edwards M. A. Lead Release to Drinking Water from Galvanized Steel Pipe Coatings. Environ. Eng. Sci. 2015, 32 (8), 713–721. 10.1089/ees.2015.0073.

[ref62] Ramamurthy T.; Ghosh A.; Pazhani G. P.; Shinoda S. Current Perspectives on Viable but Non-Culturable (VBNC) Pathogenic Bacteria. Front. Public Health 2014, 2, 103 10.3389/fpubh.2014.00103. 25133139 PMC4116801

[ref63] Declerck P.; Behets J.; Margineanu A.; van Hoef V.; De Keersmaecker B.; Ollevier F. Replication of Legionella Pneumophila in Biofilms of Water Distribution Pipes. Microbiol. Res. 2009, 164 (6), 593–603. 10.1016/j.micres.2007.06.001. 17644359

[ref64] Moritz M. M.; Flemming H.-C.; Wingender J. Integration of Pseudomonas Aeruginosa and Legionella Pneumophila in Drinking Water Biofilms Grown on Domestic Plumbing Materials. Int. J. Hyg. Environ. Health 2010, 213 (3), 190–197. 10.1016/j.ijheh.2010.05.003. 20556878

[ref65] Alleron L.; Khemiri A.; Koubar M.; Lacombe C.; Coquet L.; Cosette P.; Jouenne T.; Frere J. VBNC Legionella Pneumophila Cells Are Still Able to Produce Virulence Proteins. Water Res. 2013, 47 (17), 6606–6617. 10.1016/j.watres.2013.08.032. 24064547

[ref66] Dowdell K.; Haig S.-J.; Caverly L. J.; Shen Y.; LiPuma J. J.; Raskin L. Nontuberculous Mycobacteria in Drinking Water Systems – The Challenges of Characterization and Risk Mitigation. Curr. Opin. Biotechnol. 2019, 57, 127–136. 10.1016/j.copbio.2019.03.010. 31003169 PMC6924000

[ref67] Standard Test Method for Determining the Antimicrobial Activity of Antimicrobial Agents Under Dynamic Contact Conditions. https://www.astm.org/e2149-20.html (accessed 2023-07-05).

[ref68] Berry D.; Xi C.; Raskin L. Microbial Ecology of Drinking Water Distribution Systems. Curr. Opin. Biotechnol. 2006, 17 (3), 297–302. 10.1016/j.copbio.2006.05.007. 16701992

[ref69] Schwartz T.; Kohnen W.; Jansen B.; Obst U. Detection of Antibiotic-Resistant Bacteria and Their Resistance Genes in Wastewater, Surface Water, and Drinking Water Biofilms. FEMS Microbiol. Ecol. 2003, 43 (3), 325–335. 10.1111/j.1574-6941.2003.tb01073.x. 19719664

[ref70] Mullis S. N.; Falkinham J. O. Adherence and Biofilm Formation of Mycobacterium Avium, Mycobacterium Intracellulare and Mycobacterium Abscessus to Household Plumbing Materials. J. Appl. Microbiol. 2013, 115 (3), 908–914. 10.1111/jam.12272. 23742161

[ref71] Neu L.; Hammes F. Feeding the Building Plumbing Microbiome: The Importance of Synthetic Polymeric Materials for Biofilm Formation and Management. Water 2020, 12 (6), 1774 10.3390/w12061774.

[ref72] Yonathan K.; Mann R.; Mahbub K. R.; Gunawan C. The Impact of Silver Nanoparticles on Microbial Communities and Antibiotic Resistance Determinants in the Environment. Environ. Pollut. 2022, 293, 118506 10.1016/j.envpol.2021.118506. 34793904

[ref73] Vaz-Moreira I.; Nunes O. C.; Manaia C. M. Ubiquitous and Persistent Proteobacteria and Other Gram-Negative Bacteria in Drinking Water. Sci. Total Environ. 2017, 586, 1141–1149. 10.1016/j.scitotenv.2017.02.104. 28238372

[ref74] Pinto A. J.; Xi C.; Raskin L. Bacterial Community Structure in the Drinking Water Microbiome Is Governed by Filtration Processes. Environ. Sci. Technol. 2012, 46 (16), 8851–8859. 10.1021/es302042t. 22793041

[ref75] El-Chakhtoura J.; Saikaly P. E.; van Loosdrecht M. C. M.; Vrouwenvelder J. S. Impact of Distribution and Network Flushing on the Drinking Water Microbiome. Front. Microbiol. 2018, 9, 2205 10.3389/fmicb.2018.02205. 30283424 PMC6157312

[ref76] Mi Z.; Dai Y.; Xie S.; Chen C.; Zhang X. Impact of Disinfection on Drinking Water Biofilm Bacterial Community. J. Environ. Sci. 2015, 37, 200–205. 10.1016/j.jes.2015.04.008. 26574105

[ref77] Jia S.; Shi P.; Hu Q.; Li B.; Zhang T.; Zhang X.-X. Bacterial Community Shift Drives Antibiotic Resistance Promotion during Drinking Water Chlorination. Environ. Sci. Technol. 2015, 49 (20), 12271–12279. 10.1021/acs.est.5b03521. 26397118

[ref78] Delafont V.; Bouchon D.; Héchard Y.; Moulin L. Environmental Factors Shaping Cultured Free-Living Amoebae and Their Associated Bacterial Community within Drinking Water Network. Water Res. 2016, 100, 382–392. 10.1016/j.watres.2016.05.044. 27219048

[ref79] Farhat M.; Alkharsah K. R.; Alkhamis F. I.; Bukharie H. A. Metagenomic Study on the Composition of Culturable and Non-Culturable Bacteria in Tap Water and Biofilms at Intensive Care Units. J. Water Health 2019, 17 (1), 72–83. 10.2166/wh.2018.213. 30758305

[ref80] Vaz-Moreira I.; Nunes O. C.; Manaia C. M. Diversity and Antibiotic Resistance Patterns of Sphingomonadaceae Isolates from Drinking Water. Appl. Environ. Microbiol. 2011, 77 (16), 5697–5706. 10.1128/AEM.00579-11. 21705522 PMC3165245

[ref81] Sun S.; Zhou Y.; Yu H.; Li W.; Zhou W.; Luo G.; Zhang W. Effect of Pipe Materials on Bacterial Community, Redox Reaction, and Functional Genes. Coatings 2022, 12 (11), 1747 10.3390/coatings12111747.

[ref82] M., Madigan; Kelly B.; Buckley D.; Matthew Sattley W.; Stahl D. Brock Biology of Microorganisms, 15th ed.; Pearson, 2019.

[ref83] Lau P. C. Y.; Lindhout T.; Beveridge T. J.; Dutcher J. R.; Lam J. S. Differential Lipopolysaccharide Core Capping Leads to Quantitative and Correlated Modifications of Mechanical and Structural Properties in Pseudomonas Aeruginosa Biofilms. J. Bacteriol. 2009, 191 (21), 6618–6631. 10.1128/JB.00698-09. 19717596 PMC2795305

[ref84] Maldonado R. F.; Sá-Correia I.; Valvano M. A. Lipopolysaccharide Modification in Gram-Negative Bacteria during Chronic Infection. FEMS Microbiol. Rev. 2016, 40 (4), 480–493. 10.1093/femsre/fuw007. 27075488 PMC4931227

[ref85] Formosa-Dague C.; Castelain M.; Martin-Yken H.; Dunker K.; Dague E.; Sletmoen M. The Role of Glycans in Bacterial Adhesion to Mucosal Surfaces: How Can Single-Molecule Techniques Advance Our Understanding?. Microorganisms 2018, 6 (2), 39 10.3390/microorganisms6020039. 29734645 PMC6027152

[ref86] Landrygan-Bakri J.; Wilson M. J.; Williams D. W.; Lewis M. A. O.; Waddington R. J. Real-Time Monitoring of the Adherence of *Streptococcus Anginosus* Group Bacteria to Extracellular Matrix Decorin and Biglycan Proteoglycans in Biofilm Formation. Res. Microbiol. 2012, 163 (6–7), 436–447. 10.1016/j.resmic.2012.07.006. 22835945

[ref87] Wong E. H. J.; Ng C. G.; Goh K. L.; Vadivelu J.; Ho B.; Loke M. F. Metabolomic Analysis of Low and High Biofilm-Forming Helicobacter Pylori Strains. Sci. Rep. 2018, 8 (1), 1409 10.1038/s41598-018-19697-0. 29362474 PMC5780479

[ref88] Chu E. K.; Kilic O.; Cho H.; Groisman A.; Levchenko A. Self-Induced Mechanical Stress Can Trigger Biofilm Formation in Uropathogenic Escherichia Coli. Nat. Commun. 2018, 9, 4087 10.1038/s41467-018-06552-z. 30291231 PMC6173693

[ref89] Fazeli-Nasab B.; Sayyed R. Z.; Mojahed L. S.; Rahmani A. F.; Ghafari M.; Antonius S.; Sukamto Biofilm production: A strategic mechanism for survival of microbes under stress conditions. Biocatal. Agric. Biotechnol. 2022, 42, 102337 10.1016/j.bcab.2022.102337.

[ref90] Stabryla L. M.; Johnston A.; Millstone J. E.; Gilbertson M. Emerging investigator series: it’s not all about the ion: support for particle-specific contributions to silver nanoparticle antimicrobial activity. Environ. Sci. Nano 2018, 5 (9), 2047–2068. 10.1039/C8EN00429C.

[ref91] Stüken A.; Haverkamp T. H. A.; Dirven H. A. A. M.; Gilfillan G. D.; Leithaug M.; Lund V. Microbial Community Composition of Tap Water and Biofilms Treated with or without Copper–Silver Ionization. Environ. Sci. Technol. 2018, 52 (6), 3354–3364. 10.1021/acs.est.7b05963. 29461810

[ref92] Li N.; Li X.; Shi Z.-Y.; Fan X.-Y.; Zhou Z.-W. Bacterial Communities, Potential Pathogens and Antibiotic Resistance Genes of Silver-Loaded Stainless Steel Pipe Wall Biofilm in Domestic Hot Water System. J. Water Process Eng. 2021, 40, 101935 10.1016/j.jwpe.2021.101935.

[ref93] Albalghiti E.; Stabryla L. M.; Gilbertson L. M.; Zimmerman J. B. Towards Resolution of Antibacterial Mechanisms in Metal and Metal Oxide Nanomaterials: A Meta-Analysis of the Influence of Study Design on Mechanistic Conclusions. Environ. Sci. Nano 2021, 8 (1), 37–66. 10.1039/D0EN00949K.

[ref94] Forstner C.; Orton T. G.; Wang P.; Kopittke P. M.; Dennis P. G. Wastewater Treatment Processing of Silver Nanoparticles Strongly Influences Their Effects on Soil Microbial Diversity. Environ. Sci. Technol. 2020, 54 (21), 13538–13547. 10.1021/acs.est.0c01312. 33052663

[ref95] Wang H.; Min C.; Xia F.; Xia Y.; Tang M.; Li J.; Hu Y.; Zou M. Metagenomic Analysis Reveals the Short-Term Influences on Conjugation of blaNDM-1 and Microbiome in Hospital Wastewater by Silver Nanoparticles at Environmental-Related Concentration. Environ. Res. 2023, 228, 115866 10.1016/j.envres.2023.115866. 37037312

[ref96] Przemieniecki S. W.; Oćwieja M.; Ciesielski S.; Halecki W.; Matras E.; Gorczyca A. Chemical Structure of Stabilizing Layers of Negatively Charged Silver Nanoparticles as an Effector of Shifts in Soil Bacterial Microbiome under Short-Term Exposure. Int. J. Environ. Res. Public. Health 2022, 19 (21), 14438 10.3390/ijerph192114438. 36361318 PMC9658158

[ref97] Li X.; Wang H.; Hu C.; Yang M.; Hu H.; Niu J. Characteristics of Biofilms and Iron Corrosion Scales with Ground and Surface Waters in Drinking Water Distribution Systems. Corros. Sci. 2015, 90, 331–339. 10.1016/j.corsci.2014.10.028.

[ref98] Kimbell L. K.; Wang Y.; McNamara P. J. The Impact of Metal Pipe Materials, Corrosion Products, and Corrosion Inhibitors on Antibiotic Resistance in Drinking Water Distribution Systems. Appl. Microbiol. Biotechnol. 2020, 104 (18), 7673–7688. 10.1007/s00253-020-10777-8. 32734389

[ref99] Sun H.; Shi B.; Lytle D. A.; Bai Y.; Wang D. Formation and Release Behavior of Iron Corrosion Products under the Influence of Bacterial Communities in a Simulated Water Distribution System. Environ. Sci. Process. Impacts 2014, 16 (3), 576–585. 10.1039/c3em00544e. 24509822

[ref100] Donohue M. J. Epidemiological Risk Factors and the Geographical Distribution of Eight Mycobacterium Species. BMC Infect. Dis. 2021, 21 (1), 258 10.1186/s12879-021-05925-y. 33706712 PMC7953749

[ref101] Falkinham J. O. Current Epidemiologic Trends of the Nontuberculous Mycobacteria (NTM). Curr. Environ. Health Rep 2016, 3 (2), 161–167. 10.1007/s40572-016-0086-z. 27020801

[ref102] Prabaker K.; Muthiah C.; Hayden M. K.; Weinstein R. A.; Cheerala J.; L. Scorza M.; Segreti J.; Lavin M. A.; Schmitt B. A.; Welbel S. F.; Beavis K. G.; Trenholme G. M. Pseudo-Outbreak of *Mycobacterium Gordonae* Following the Opening of a Newly Constructed Hospital at a Chicago Medical Center. Infect. Control Hosp. Epidemiol. 2015, 36 (2), 198–203. 10.1017/ice.2014.28. 25633003

[ref103] Brown-Elliott B. A.; Philley J. V. Rapidly Growing Mycobacteria. Microbiol. Spectr. 2017, 5 (1), 28084211 10.1128/microbiolspec.tnmi7-0027-2016. PMC11687460 28084211

[ref104] Yang J.; Hu Y.; Zhang Y.; Zhou S.; Meng D.; Xia S.; Wang H. Deciphering the Diversity and Assemblage Mechanisms of Nontuberculous Mycobacteria Community in Four Drinking Water Distribution Systems with Different Disinfectants. Sci. Total Environ. 2024, 907, 168176 10.1016/j.scitotenv.2023.168176. 37907107

[ref105] Boopathi S.; Ramasamy S.; Haridevamuthu B.; Murugan R.; Veerabadhran M.; Jia A.-Q.; Arockiaraj J. Intercellular Communication and Social Behaviors in Mycobacteria. Front. Microbiol. 2022, 13, 943278 10.3389/fmicb.2022.943278. 36177463 PMC9514802

[ref106] Haig S.-J.; Kotlarz N.; LiPuma J. J.; Raskin L. A High-Throughput Approach for Identification of Nontuberculous Mycobacteria in Drinking Water Reveals Relationship between Water Age and Mycobacterium Avium. mBio 2018, 9 (1), e02354 10.1128/mBio.02354-17. 29440575 PMC5821076

