# Supplementary material for: Not the Silver Bullet: Uncovering the Unexpected Limited Impacts of Silver-Containing Showerheads on the Drinking Water Microbiome
Source: ACS ES T Water. 2024 Nov 25;4(12):5364–76. doi: 10.1021/acsestwater.4c00492 (PMC11650587; doi:10.1021/acsestwater.4c00492)
Supplement: Supplementary file 1 — ew4c00492_si_001.pdf [file ew4c00492_si_001.pdf]

Supplementary Information

Not the silver bullet: Uncovering the unexpected limited impacts of silver-containing showerheads on the drinking water microbiome

Sarah Pitell[1], Isaiah Spencer-Williams[1], Daniel Huffman[1], Paige Moncure[2], Jill Millstone[2,3,4], Janet Stout[1,5], Leanne Gilbertson[1,4§], Sarah-Jane Haig[1,6]*

[1]*Department of Civil and Environmental Engineering, University of Pittsburgh, Pennsylvania, USA*
[2]*Department of Chemistry, University of Pittsburgh, Pennsylvania, USA*
[3]*Department of Mechanical Engineering and Materials Science, University of Pittsburgh, Pittsburgh, Pennsylvania 15260, USA*
[4]*Department of Chemical and Petroleum Engineering, University of Pittsburgh, Pennsylvania, USA*
[5]*Special Pathogens Laboratory, Pittsburgh, Pennsylvania, USA*
[6]*Department of Environmental & Occupational Health, University of Pittsburgh, Pennsylvania, USA*
**Corresponding author: sjhaig@pitt.edu*

Summary of contents: 13 Pages, 5 Tables, 5 Figures

TABLE OF CONTENTS

Table A1. Different physical and chemical water quality parameters measured in this study and corresponding analytical method used.

Table A2. Molecular primers, thresholds, and assay sensitivity for ddPCR analysis.

Table A3. Average values of measured water chemistry parameters.

Table A4: Summary of generated linear models in water samples. In the model components column, ± indicates positive or negative association and the percent of the variance explained by each variable is superscripted.

Table A5: Average diversity values of water samples ± standard deviation. Sample size is denoted in parentheses under value.

Figure A1. Schematic of full-scale shower laboratory set-up. The blue X indicates the aerosol sampling port in the Plexiglass door, the black X indicates where the copper pipe from the hot water heater connects to the shower stall, and the red X's correspond to each showerhead position.

Figure A2: Summary of SEM-EDS analysis conducted on silver-containing inserts of the silver-embedded head A. Annotated diagram of the sampling sites of the shower insert, with A1 being the 'top' of the insert, A2 being taken from the side wall, and A3 being the 'bottom' of the insert. B. Representative EDS scan from an insert with red corresponding to sampling site A1, blue

corresponding to sampling site A2, and yellow corresponding to sampling site A3. C. Representative SEM image taken from the showerhead insert, with maps of i. oxygen and ii. titanium.

Figure A3: Non-metric Multidimensional Scaling plot of water samples by showerhead type

Figure A4: Shannon diversity calculated from water samples by showerhead type.

Figure A5: Viable concentrations of A. *L. pneumophila*, B. *P. aeruginosa*, C. *M. gordonae*, D. NTM, E. total bacteria, and F. *M. mucogenicum/phocaicum* for ABS plastic (red), metal (yellow), silver-coated copper mesh (green), silver-embedded (blue), and silver mesh (purple) showerheads by biofilm formation stage. Significant differences are marked with a colored bracket and star corresponding to the showerhead type and p-value (* = $p \leq 0.05$, ** = $p \leq 0.01$, *** = $p \leq 0.001$).

Supplementary Information

Supplementary Tables

Table A1. Different physical and chemical water quality parameters measured in this study and corresponding analytical method used.

Parameter	Units	Analytical Technique	LOD/LOQ
**Temperature**	°C	Thermometer	
**pH**		pH electrode	
**Free Chlorine**	mg/L as Cl2	DPD method	0.01/0.02
**Total Chlorine**	mg/L as Cl2	DPD method	0.01/0.02
**Ammonia**	mg/L as NH3-N	Salicylate method	0.01/0.01
**Orthophosphate**	mg/L as $PO4^{3-}$	Ascorbic acid method	0.01/0.02
**Total Organic Carbon**	mg/L	TOC analyzer	0.01/0.01
**Dissolved Organic Carbon**	mg/L	TOC analyzer (0.45 µm filtered)	0.01/0.01
**Total Iron**	µg/L	ICP-MS	0.01/0.01
**Total Copper**	µg/L	ICP-MS	0.01/0.01
**Total Silver**	µg/L	ICP-MS	0.01/0.01
**Total Lead**	µg/L	ICP-MS	0.01/0.01
**Total Calcium**	µg/L	ICP-MS	0.01/0.01
**Total Magnesium**	µg/L	ICP-MS	0.01/0.01
**Total Cadmium**	µg/L	ICP-MS	0.01/0.01
**Total Zinc**	µg/L	ICP-MS	0.01/0.01
**Dissolved Iron**	µg/L	ICP-MS (0.45 µm filtered)	0.01/0.01
**Dissolved Copper**	µg/L	ICP-MS (0.45 µm filtered)	0.01/0.01
**Dissolved Silver**	µg/L	ICP-MS (0.45 µm filtered)	0.01/0.01
**Dissolved Lead**	µg/L	ICP-MS (0.45 µm filtered)	0.01/0.01
**Dissolved Calcium**	µg/L	ICP-MS (0.45 µm filtered)	0.01/0.01
**Dissolved Magnesium**	µg/L	ICP-MS (0.45 µm filtered)	0.01/0.01
**Dissolved Cadmium**	µg/L	ICP-MS (0.45 µm filtered)	0.01/0.01
**Dissolved Zinc**	µg/L	ICP-MS (0.45 µm filtered)	0.01/0.01

Supplementary Information

Table A2. Molecular primers, thresholds, and assay sensitivity for ddPCR analysis.

Target Species	Forward (5'-3')	Reverse (5'-3')	Approx. Amplicon Size (bp)	Ref	Limit of Detection and Quantification (copies/20 µL)	Threshold for water samples
***Legionella pneumophila Lmip* gene**	*LpneuF* CCGATGCCACATCATAGC	*LpneuR* CCAATTGAGCGCCACTCATAG	150	1	6.08	7793
***Pseudomonas aeruginosa Orpl* gene**	Ps-F CGAGTACAACATGGCTCTGG	Ps-R ACCGGACGCTCTTTACCATA	117	2	7.3	5560
***Nontuberculous mycobacteria atpE* gene**	FatpE CGGYGCCGGTATCGGYGA	RatpE CGAAGACGAACARSGCCAT	164	3	5.6	9800
***Total bacteria 16s rRNA* gene**	Eub338 ACTCCTACGGGAGGCAG	Eub518 ATTACCGCGGCTGCTGG	200	4	5.3	9500

	Thermocycling Conditions
L. pneumophila* and *P. aeruginosa	95 °C for 5 min, [95 °C for 1 min, 56 °C for 1 min, 72 °C for 2 min] x 45, 4 °C for 5 min, 90 °C for 5 min
**NTM**	95 °C for 5 min, [95 °C for 1 min, 59 °C for 1 min, 72 °C for 2 min] x 45, 4 °C for 5 min, 90 °C for 5 min
**Total bacteria**	95 °C for 5 min, [95 °C for 1 min, 60 °C for 1 min, 72 °C for 2 min] x 45, 4 °C for 5 min, 90 °C for 5 min

References: 1. Wullings, B. A. et al. 2011. Appl Environ Microbiol 77 (2), 634-641 2. Feizabadi MM et al. 2010. Infect Genet Evol 10: 1247-1251 3. Radomski, N., et al., 2013. BMC Microbiol, 13(1), 277 4. Fierer, N., et al., 2005. App, Env, Microbiol, 71(7), 4117-4120

Supplementary Information

Table A3. Average values of measured water chemistry parameters.

Showerhead type	ABS Plastic	Metal	Silver Mesh	Silver-coated Copper Mesh	Silver-embedded
**Temperature (°C)**	24.7 ± 2.6	24.6 ± 3.6	24.8 ± 2.5	23.7 ± 2.3	25.3 ± 2.8
**pH**	7.3 ± 0.6	7.3 ± 0.7	7.4 ± 0.4	7.4 ± 0.5	7.3 ± 0.5
**ORP (mV)**	222.4 ± 99.0	179.6 ± 44.6	269.7 ± 106.5	235.2 ± 89.4	181.2 ± 35.1
**Free Chlorine (mg/L)**	0.01 ± 0.01	0.01 ± 0.02	0.02 ± 0.02	0.02 ± 0.02	0.01 ± 0.01
**Total Chlorine (mg/L)**	0.02 ± 0.03	0.01 ± 0.01	0.04 ± 0.03	0.03 ± 0.04	0.01 ± 0.01
**Orthophosphate (mg/L)**	1.1 ± 0.2	1.1 ± 0.2	1.1 ± 0.2	1.1 ± 0.1	1.0 ± 0.1
**Total Carbon (mg/L)**	10.3 ± 2.1	11.0 ± 0.8	10.4 ± 1.4	10.5 ± 1.7	11.0 ± 0.9
**Inorganic Carbon (mg/L)**	9.0 ± 1.9	9.6 ± 0.8	9.2 ± 1.3	9.2 ± 1.3	9.5 ± 0.8
**Total Organic Carbon (mg/L)**	1.2 ± 0.5	1.4 ± 0.3	1.3 ± 0.5	1.3 ± 0.6	1.5 ± 0.4
**Total Dissolved Carbon (mg/L)**	10.5 ± 1.2	10.8 ± 0.9	10.3 ± 1.2	10.4 ± 1.5	10.8 ± 0.9
**Dissolved Inorganic Carbon (mg/L)**	9.4 ± 1.1	9.6 ± 0.8	9.2 ± 1.3	9.2 ± 1.4	9.5 ± 0.8
**Dissolved Organic Carbon (mg/L)**	1.1 ± 0.4	1.2 ± 0.4	1.1 ± 0.4	1.2 ± 0.5	1.3 ±0.3
**Total Silver (mg/L)**	3.3×10^{-3} ± 0.01	5.6×10^{-4} ± 2.4×10^{-3}	8.9×10^{-3} ± 0.02	0.1 ± 0.2	0.01 ± 0.02
**Total Magnesium (mg/L)**	3.9×10^{3} ± 625.6	4.0×10^{3} ± 664.0	3.9×10^{3} ± 791.3	3.9×10^{3} ± 802.2	3.9×10^{3} ± 636.5
**Total Copper (mg/L)**	59.1 ± 36.3	86.5 ± 36.1	45.7 ± 22.3	101.3 ± 78.6	88.7 ± 21.8
**Total Iron (mg/L)**	248.8 ± 463.2	440.1 ± 674.7	51.4 ± 14.2	52.7 ± 12.1	417.0 ± 599.5
**Total Lead (mg/L)**	0.2 ± 0.3	1.4 ± 1.0	0.3 ± 0.5	0.2 ± 0.2	0.4 ± 0.4
**Total Zinc (mg/L)**	77.9 ± 47.8	121.5 ± 27.4	55.8 ± 58.2	45.2 ± 38.8	118.6 ± 43.8
**Total Manganese (mg/L)**	5.0 ± 13.8	11.4 ± 24.0	0.7 ± 0.8	0.7 ± 0.8	5.7 ± 10.6
**Total Cadmium (mg/L)**	0.02 ± 0.1	9.0×10^{-3} ± 7.4×10^{-3}	3.3×10^{-3} ± 0.02	5.0×10^{-3} ± 0.02	4.5×10^{-3} ± 7.9×10^{-3}
**Dissolved Silver**	0 ± 0.01	0 ± 0	0.02 ± 0.07	0.1 ± 0.1	0.1 ± 0.4

**(mg/L)**					
**Dissolved Magnesium (mg/L)**	3.8 x 10^3 ± 608.5	3.9 x 10^3 ± 609.5	3.9 x 10^3 ± 700.6	3.9 x 10^3 ± 740.0	3.8 x 10^3 ± 583.5
**Dissolved Copper (mg/L)**	41.9 ± 31.7	63.2 ± 34.8	28.0 ±14.0	56.5 ± 28.0	67.8 ± 27.0
**Dissolved Iron (mg/L)**	186.0 ± 385.6	320.8 ± 516.5	61.5 ± 50.0	86.7 ± 136.2	307.9 ± 513.2
**Dissolved Lead (mg/L)**	0.02 ± 0.06	0.6 ± 0.5	0.2 ± 0.7	0 ± 0.06	0.1 ± 0.1
**Dissolved Zinc (mg/L)**	55.2 ± 41.6	99.0 ± 29.7	30.7 ± 30.0	25.4 ± 19.6	91.3 ± 31.9
**Dissolved Manganese (mg/L)**	5.3 ± 22.8	1.7 ± 3.0	0.6 ± 2.0	1.8 ± 5.3	0.6 ± 0.6
**Dissolved Cadmium (mg/L)**	0 ± 0.01	0.09 ± 0.3	5.6 x 10^{-3} ± 0.02	0.01 ± 0.05	2.8 x 10^{-3} ± 4.6 x 10^{-3}

Table A4: Summary of generated linear models in water samples. In the model components column, ± indicates positive or negative association and the percent of the variance explained by each variable is superscripted.

Model (Transformation)	Model Components	Overall Model Explained (%)	Overall Model *p*-value
L. pneumophila **(logarithmic)**	-Biofilm Age$^{1.8\%}$, - Stall$^{5.5\%}$, -pH$^{2.5\%}$, -Orthophosphate$^{1.9\%}$	11.9	0.01
P. aeruginosa **(logarithmic)**	-Biofilm Age$^{3.1\%}$, -Campaign$^{5.1\%}$, -Viable *L. pneumophila*$^{2.3\%}$, -Cultured *M. mucogenicum/phocaicum*$^{2\%}$	12.5	0.008
**Nontuberculous mycobacteria (logarithmic)**	-Campaign$^{2.8\%}$, -Viable *P. aeruginosa*$^{2.7\%}$, -Viable total bacteria$^{13.2\%}$, -Orthophosphate$^{6\%}$	24.7	$6.2\text{x}10^{-6}$
**Total bacteria (logarithmic)**	-Temperature$^{0.7\%}$, -Dissolved Magnesium$^{4.4\%}$, -Dissolved Copper$^{8\%}$, -Viable nontuberculous mycobacteria$^{12.2\%}$, -Campaign$^{5.4\%}$	30.7	$3.8\text{x}10^{-7}$
**Microbial community (Hellinger)**	-Total Copper$^{1.5\%}$, -Stall$^{1.9\%}$, -Total Zinc$^{2.5\%}$, -Showerhead Type$^{10.2\%}$, -Biofilm Age$^{5\%}$	21.1	

Table A5: Average diversity values of water samples ± standard deviation. Sample size is denoted in parentheses under value.

Diversity Metric		Biofilm Age		Showerhead Type	
**Richness**	29.2 ± 47 (n=54)	Initial (Days 0-13)	28.1 ± 20.9 (n=9)	ABS Plastic	21 ± 10.4 (n=36)
				Metal	29 ± 4.6 (n=18)
		Early (Days 14-42)	26 ± 54.2 (n=18)	Silver-embedded	33.4 ± 16.4 (n=18)
				Silver-coated Copper Mesh	52.3 ± 109.3 (n=18)
		Mature (Days 43-70)	34.6 ± 44.4 (n=18)	Silver Mesh	19.2 ± 21.2 (n=18)
**Evenness**	0.92 ± 0.05 (n=54)	Initial (Days 0-13)	0.92 ± 0.05 (n=9)	ABS Plastic	0.92 ± 0.06 (n=36)
				Metal	0.9 ± 0.04 (n=18)
		Early (Days 14-42)	0.93 ± 0.04 (n=18)	Silver-embedded	0.9 ± 0.03 (n=18)
				Silver-coated Copper Mesh	0.93 ± 0.06 (n=18)
		Mature (Days 43-70)	0.89 ± 0.07 (n=18)	Silver Mesh	0.93 ± 0.06 (n=18)
**Diversity**	2.8 ± 0.7 (n=54)	Initial (Days 0-13)	2.9 ± 0.5 (n=9)	ABS Plastic	2.7 ± 0.4 (n=36)
				Metal	3.0 ± 0.2 (n=18)
		Early (Days 14-42)	2.7 ± 0.6 (n=18)	Silver-embedded	3.1 ± 0.4 (n=18)
				Silver-coated Copper Mesh	2.8 ± 1.1 (n=18)
		Mature (Days 43-70)	2.9 ± 0.6 (n=18)	Silver Mesh	2.5 ± 0.5 (n=18)

Supplementary Information

Supplementary Information

Supplementary Figures

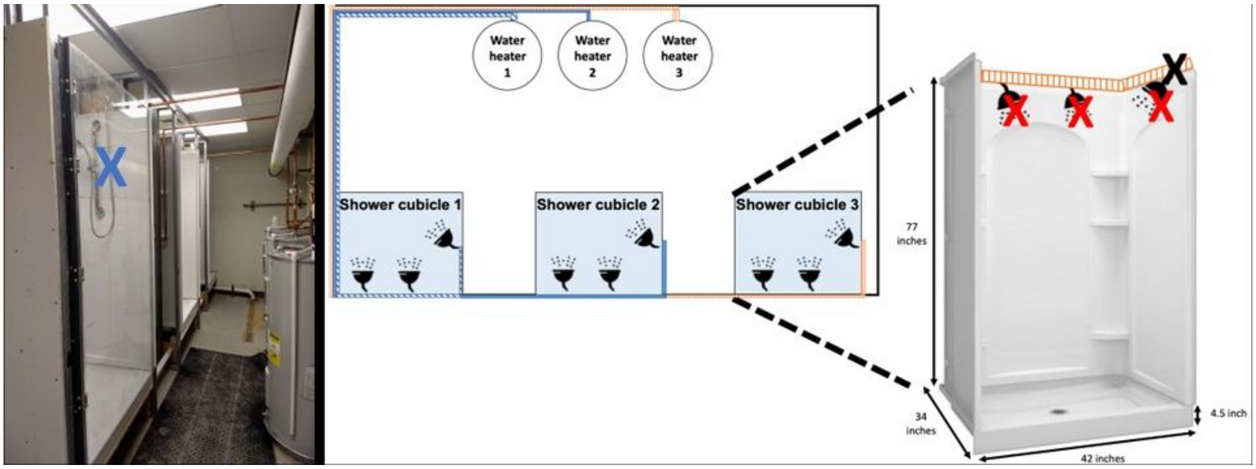

Figure A1. Schematic of full-scale shower laboratory set-up. The blue X indicates the aerosol sampling port in the Plexiglass door, the black X indicates where the copper pipe from the hot water heater connects to the shower stall, and the red X's correspond to each showerhead position.

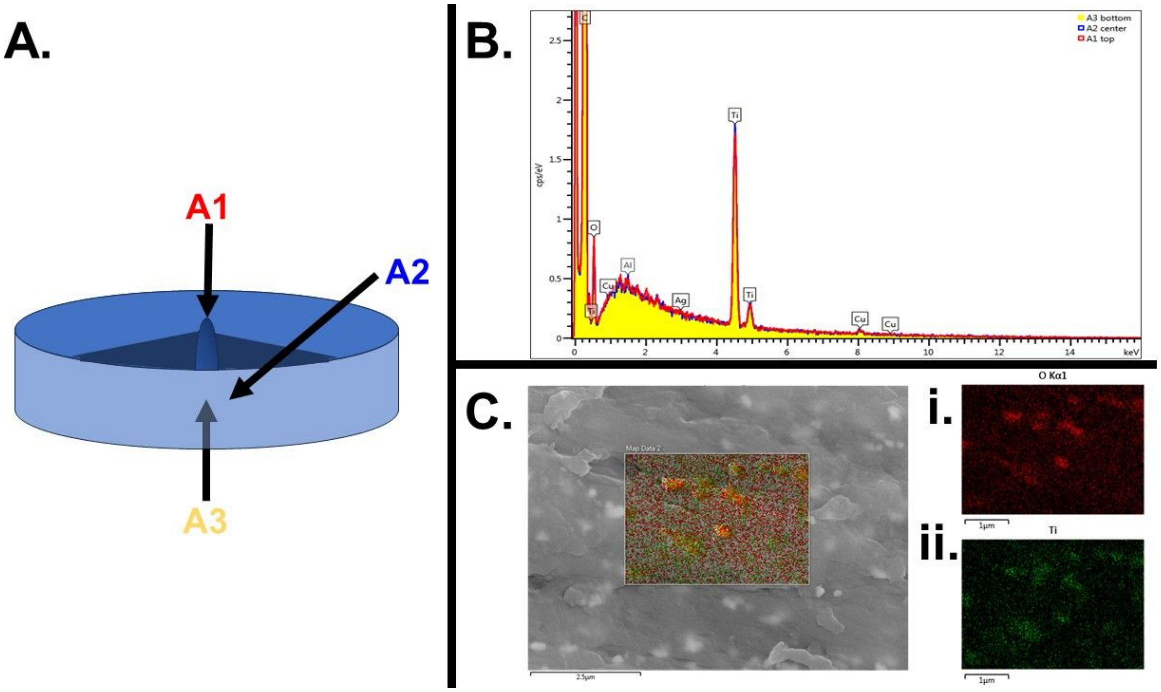

Figure A2: Summary of SEM-EDS analysis conducted on silver-containing inserts of the silver-embedded head A. Annotated diagram of the sampling sites of the shower insert, with A1 being the 'top' of the insert, A2 being taken from the side wall, and A3 being the 'bottom' of the insert. B. Representative EDS scan from an insert with red corresponding to sampling site A1, blue corresponding to sampling site A2, and yellow corresponding to sampling site A3. C. Representative SEM image taken from the showerhead insert, with maps of i. oxygen and ii. titanium.

Supplementary Information

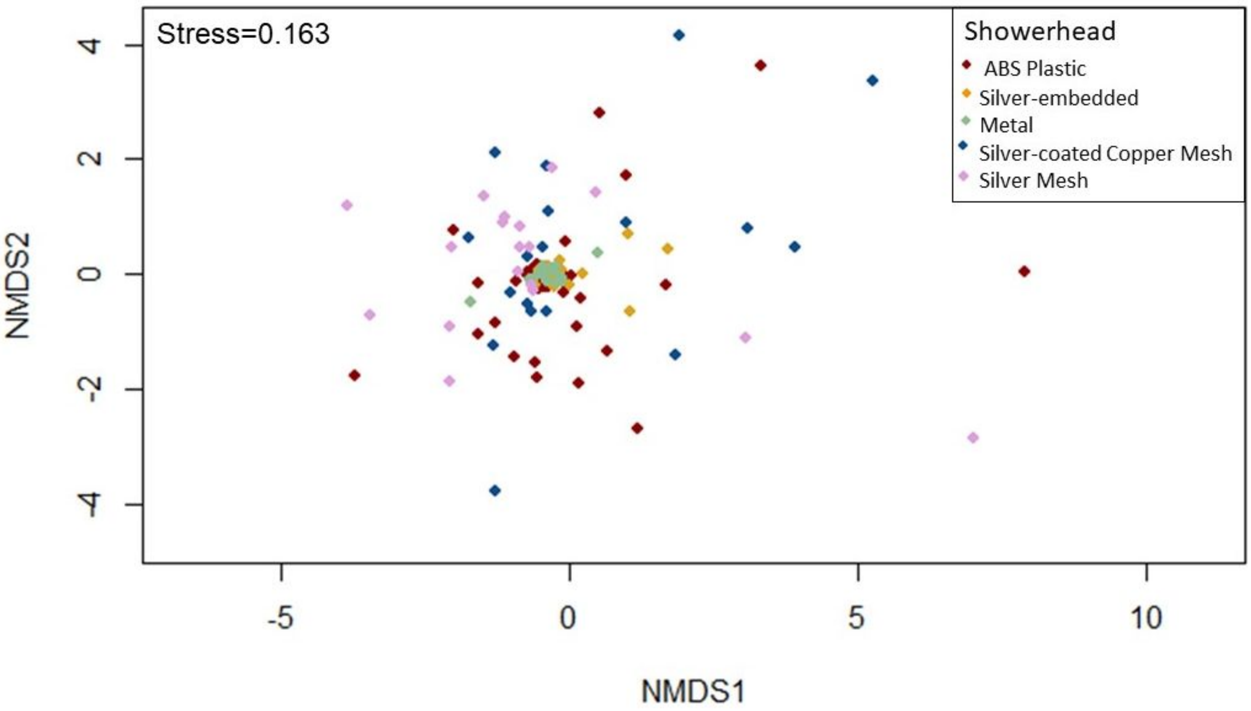

Figure A3: Non-metric Multidimensional Scaling plot of water samples by showerhead type.

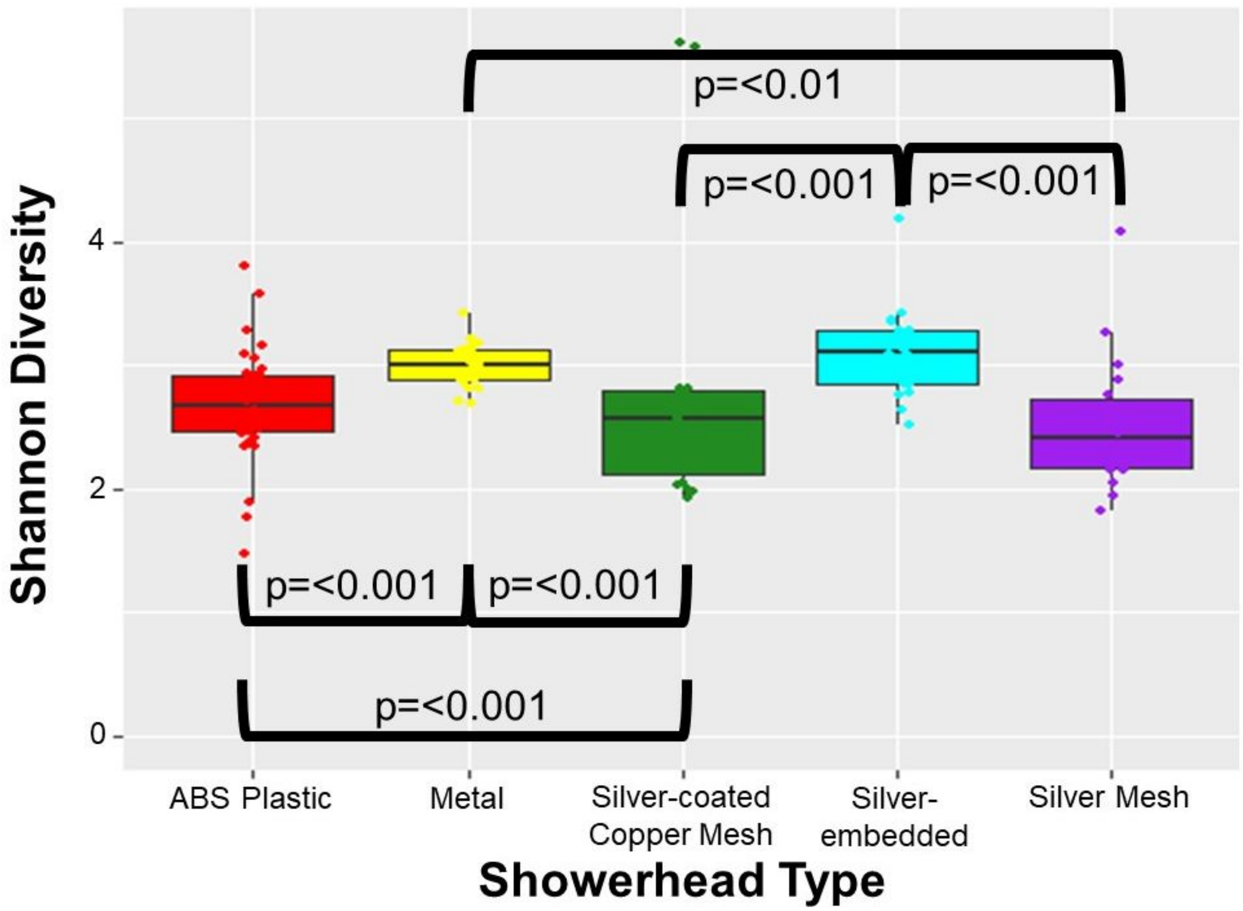

Figure A4: Shannon diversity calculated from water samples by showerhead type.

Supplementary Information

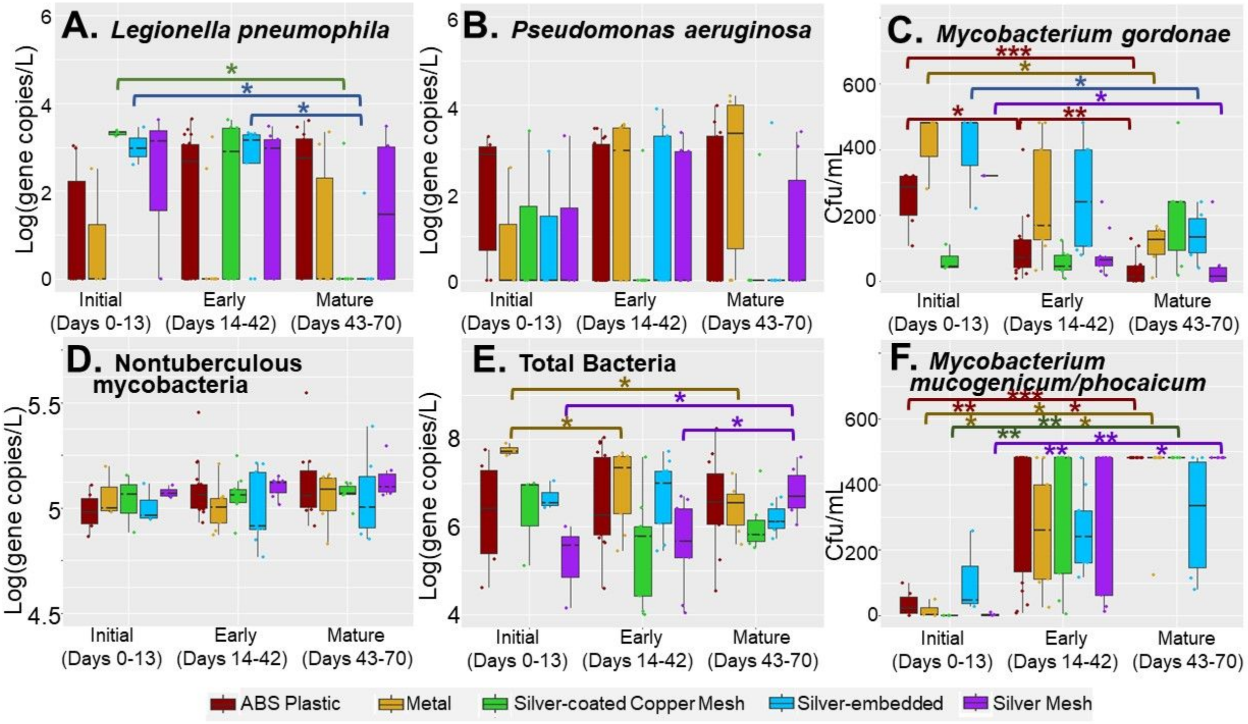

Figure A5: Viable concentrations of A. *L. pneumophila*, B. *P. aeruginosa*, C. *M. gordonae*, D. NTM, E. total bacteria, and F. *M. mucogenicum/phocaicum* for ABS plastic (red), metal (yellow), silver-coated copper mesh (green), silver-embedded (blue), and silver mesh (purple) showerheads by biofilm formation stage. Significant differences are marked with a colored bracket and star corresponding to the showerhead type and p-value (* = $p \leq 0.05$, ** = $p \leq 0.01$, *** = $p \leq 0.001$).
